# *RARRES2* is involved in the “lock-and-key” interactions between osteosarcoma stem cells and tumor-associated macrophages

**DOI:** 10.1038/s41598-024-52738-5

**Published:** 2024-01-27

**Authors:** Jingjin Ma, Zhiyu Chen, Qiaochu Li, Linbang Wang, Jiaxing Chen, Xinyu Yang, Chaohua Yang, Zhengxue Quan

**Affiliations:** 1 Department of Orthopedics, The First Affiliated Hospital of Chongqing Medical University, Chongqing, 400016 China; 2 Department of Orthopedics, Peking University Third Hospital, Beijing, 100191 China

**Keywords:** Cancer metabolism, Sarcoma

## Abstract

Osteosarcoma (OS) is a type of tumor. Osteosarcoma stem cells (OSCs) are responsible for drug resistance, recurrence, and immunosuppression in OS. We aimed to determine the heterogeneity of OSCs and the immunosuppression mechanisms underlying the interactions between OSCs and tumor-associated macrophages (TAMs). The cell components, trajectory changes, and cell communication profiles of OS cells were analyzed by transcriptomics at the single-cell level. The intercellular communication patterns of OSCs were verified, and the role of the cell hub genes was revealed. Hub geneS are genes that play important roles in regulating certain biological processes; they are often defined as the genes with the strongest regulatory effect on differentially expressed gene sets. Moreover, various cellular components of the OS microenvironment were identified. Malignant cells were grouped, and OSCs were identified. Further regrouping and communication analysis revealed that the genes in the stemness maintenance and differentiation subgroups were involved in communication with macrophages. Key receptor–ligand pairs and target gene sets for cell communication were obtained. Transcriptome data analysis revealed the key gene *RARRES2,* which is involved in intercellular communication between OSCs and TAMs. In vitro studies confirmed that macrophages promote *RARRES2*-mediated stemness maintenance in OSCs via the TAM-secreted cytokine insulin-like growth factor 1. Patient studies confirmed that *RARRES2* could be a biomarker of OS. OSCs are highly heterogeneous, and different subgroups are responsible for proliferation and communication with other cells. The IGF-*RARRES2* axis plays a key role in maintaining OSC stemness through communication with TAMs.

## Introduction

Cancer stem cells (CSCs) are a subset of tumor cells that possess features similar to normal stem cells, such as self-renewal ability and multipotent differentiation potential. Osteosarcoma (OS) is a highly malignant bone tumor with a high relapse rate, and surgery and chemotherapy are the main treatment strategies for patients diagnosed with nonmetastatic OS or OS with micrometastasis^1^. However, multidrug resistance causes poor outcomes^2,3^ and can be triggered by the development of osteosarcoma stem cells (OSCs) during tumorigenesis and progression^4^. OSCs have been identified as a small group of OS cells with tumor-initiating potential, multipotency, and self-renewal ability^5,6^. Targeting OSCs may be useful for suppressing OS metastasis and overcoming multidrug resistance. However, the heterogeneity of OSCs has yet to be revealed.

Another major pathological characteristic of OS is its highly complicated tumor microenvironment (TME), which is characterized by the infiltration of malignant mesenchymal tumor cells and multiple types of immune and stromal cells^7^. Like embryonic stem cells, OSCs undergo differentiation that is influenced by external factors, such as the environment, and internal factors, such as genetics^8^. Ample evidence indicates that the biological phenotype and behavior of OSCs are profoundly affected by the TME^9^. Bidirectional interactions between OSCs and the TME in different tumor types promote tumor progression in different ways^10,11^.

As an essential component of the TME, tumor-associated macrophages (TAMs) are involved in several mechanisms underlying tumor biology^12^. Increasing evidence indicates that chronic inflammation, or even prolonged inflammatory episodes in which macrophages participate, can support the develop of a microenvironment suitable for oncogenesis^13^. Furthermore, TAMs continually contribute to chronic inflammation by secreting inflammatory cytokines, such as CXCL8, IL-1β, and IL-6^14,15^. Recent evidence suggests that TAMs are critical for the maintenance and self-renewal of cancer stem cells (CSCs) in multiple tumor types^16,17^. However, the underlying molecular mechanisms, such as immune regulations, remain unclear.

To reveal the biological characteristics of OSCs and their interactions with TAMs, we adopted an unbiased approach using scRNA-seq to identify the components of the OS microenvironment and preliminarily analyzed the communication between different cell populations. We subgrouped malignant cells to identify OSCs and further subgrouped OSCs to identify genes involved in stemness maintenance and differentiation. Subsequently, through transcriptome data analysis, we identified the key gene responsible for the communication between OSCs and TAMs.

## Methods

### Dataset acquisition

To investigate the gene expression features of OSCs at the single-cell level and their communication patterns with other TME cells, we integrated single-cell RNA sequencing (scRNA-seq) datasets from the Gene Expression Omnibus (GEO) database (https://www.ncbi.nlm.nih.gov/geo/query/acc.cgi?acc=GSE152048).

Transcriptome sequencing data for OS samples with updated clinical data were downloaded from the Therapeutically Applicable Research to Generate Effective Treatments (TARGET) database (https://ocg.cancer.gov/programs/target) and the GEO database (https://www.ncbi.nlm.nih.gov/gds/?term=GSE16088)^18^.

### Single-cell transcriptome standard analysis

The scater R package was used for quality control of the single-cell RNA-seq data^19^. The scimpute R and scran R packages were applied for imputation and normalization. Specifically, standard log normalization was conducted on multiple scRNA datasets to identify variable features of each individual^20^, and anchors between datasets were determined by the FindIntegrationAnchors function. The anchors were subsequently assessed via the IntegrateData function, returned as a Seurat object, and applied for downstream analysis. Subtypes of OS cells, OS-infiltrating immune cells, and stromal cells were classified and individually identified using SingleR21; the resolution value used in the first classification was 0.27, and the resolution value used in the second classification for analysis of OS heterogeneity was 0.27. For cell type identification, we conducted preliminary identification via singleR, and further identified the types of cell subsets with reference to the publication “Single-cell RNA landscape of intratumoral heterogeneity and immunosuppressive microenvironment in advanced osteosarcoma”. The AUCell score was used to further analyze the different biological activities of the cell clusters^21^, and the org.Hs.eg.db R package enrichplot was used for functional analysis.

### Cell trajectory analysis

The Monocle2 (version 2.4.0) algorithm was used to construct a single-cell pseudotime trajectory, and gene expression changes as the cells underwent differentiation were identified. The screening criteria for genes were as follows: genes that were expressed in ≥ 10 cells with a mean expression value of ≥ 0.05 and dispersion empirical value of ≥ 2 were selected for cell ordering.

### Cell–cell communication analysis

The interaction between tumor cells and other cell types in the OS microenvironment was investigated through ligand–receptor (LR) interaction analysis using the iTALK package. CellChat (1.1.0), a repository of ligands, receptors, and cofactors, was used to analyze the pathways involved in cell–cell communication. The regulatory gene networks of cell interactions were inferred using NicheNet^22^. NicheNet uses an incident ligand-based method to identify ligand activity in receiving cells and filters ligand candidates by processing the receiver and ligand expression from the sending cells. We performed a differential NicheNet analysis between niches of interest pipelines (https://github.com/saeyslab/nichenetr/blob/master/vignettes/differential_nichenet.md) to identify the key target genes in OSCs regulated by OSC–TAM interactions.

### Consensus clustering for OSC–TAM communication subtypes and collection of OSCs signatures

The differential expression of OSC–TAM communication-related genes (OTCRGs) between the tumor and normal groups was analyzed, and *p* < 0.05 was considered to indicate statistical significance. Consensus clustering algorithms were used for the unsupervised classification of OS samples via the ConsensusClusterPlus R package (v1.50.0) based on OTCRGs^23^. The k-means (km) cluster method was applied for this analysis with 1000 repeated iterations to ensure dependability, and the GSVA R package (v1.34.0)^24^ was applied to illustrate the differential biological functions between the two clusters. Subsequently, single sample gene set enrichment analysis (ssGSEA) was implemented to quantitatively determine the proportions of different subclusters of CSCs and TAMs in each OS sample.

### Screening and validation of diagnostic genes

Multiple machine learning algorithms, including LASSO and SVM-RFE, were used to construct predictive signatures and screen candidate diagnostic genes. LASSO is a compressive estimation approach and is also known as a biased estimator. When dealing with complex covariance in data, it creates models by building a penalty function that forces it to compress some regression coefficients. Receiver operating characteristic (ROC) curves were used to assess the predictive effects of the signatures. SVM-RFE is a backward sequential selection algorithm that applies the support vector machine principle. It trains the sample with the model, ranks each feature according to its score, removes the feature with the lowest score, and trains the model until the required number of features is chosen. The R package “glmnet” was used for LASSO analysis, and the R package “e1071” for was used for SVM-RFE. The intersecting gene of the two algorithms was deemed the candidate diagnostic gene.

### Cell culture

The human OS cell line 143B was obtained from the American Type Culture Collection and cultured as previously described. Human bone marrow (BM) aspirate samples were obtained from donors at Honghui Hospital. The study was approved by the Ethics Committee of Honghui Hospital (Approval Number: 202303051) and was conducted in accordance with the Declaration of Helsinki. Primary human BM cells (BM cells first collected by density gradient centrifugation from BM cultured in complete α-MEM) were separated from the BM^25^. Then, an auto MACS Pro Separator (Miltenyi Biotec, Bergisch Gladbach, Germany) was used to purify the CD14-positive bone marrow cells for macrophage differentiation induction by culture with human hM-CSF at a concentration of 25 ng/mL (R&D Systems) for three days^26^. Human OS cells (143B) were seeded in the lower chamber of a 24-well, 0.4-μm pore transwell system (Corning, Glendale, AZ). BM-derived macrophages were added to the upper chamber for coculture^27^.

### Screening and culturing of OSCs

The passaged 1 × 10^6^ 143B cells were first cultured in a suspension culture system containing a variety of growth factors in a stem cell-specific serum-free medium for 48 h. CD133 + cells were purified using immunomagnetic separation miniMACS (Miltenyi Biotec, Germany)^27^. After the small cell clusters aggregated and formed cell spheres, the suspension culture was continued for 10 days until the formation of typical tumorspheres.

### Sh-RARRES2 and Si-RARRES2 RNA interference

Stable cell lines were cultured in complete medium supplemented with 0.5 μg/mL puromycin. The transfection experiments were performed according to the manufacturer’s instructions with Lipofectamine 2000TM (Invitrogen).

The 143B cells were seeded in 6-well plates for 24 h to achieve 50% confluence. RARRES2 siRNA (20 nM; RiboBio, Guangzhou, China) was transfected into cells by utilizing LIPO3000 in OPTI-MEM (31985070; Thermo Fisher Scientific, Waltham, MA) according to the manufacturer’s instructions. The supernatants were removed 24 h later, and the medium was replaced with fresh medium. The cells were harvested after 72 h for further experiments. The siRNA sequences used were as follows:

Si-RARRES2-specific siRNA: AGAACUUGGGUCUCUAUGGGG and ‘Nonsense’ (control): 5′-UUCUCCGAACGUGUCACGUTT-3′.

### Cell viability assay

143B cells (1 × 10^4^) were seeded in 96-well plates and cultured with medium from TAMs for 48 h. Then, the medium was removed, and CCK-8 solution was added to each well according to the manufacturer’s protocol (Beyotime, China), followed by 4 h of incubation. The absorbance was measured at 450 nm.

### Scratch wound and transwell experiments

Confluent 143B cells were scratched using 200-µL pipette tips. The plates were washed and photographed using an inverted microscope (Olympus, Japan) at 0 h, 24 h and 36 h, after which the wound area was calculated. A transwell system was used for the cell invasion assay. The cells were seeded into the upper chamber, which was coated with an extracellular matrix (BD Biosciences, San Jose, CA). The lower chamber was filled with TAMs. After incubation, the cells under the filter were fixed and stained with crystal violet solution. The cells were photographed using a phase-contrast microscope (Olympus, Japan), and the number of cells was counted.

### Patients

OS tissues were surgically resected from 16 patients at The First Affiliated Hospital of Chongqing Medical University from January 2022 to January 2023. The inclusion criteria were a pathological diagnosis of OS and tumor resection, while the exclusion criteria were recurrence, metastasis, incomplete clinical data, and an unknown diagnosis. Adjacent tissues (n = 16) were collected from the same patients and stored in liquid nitrogen for further experiments. Informed consent was obtained from all patients in this study. This study was approved by the Ethics Committee of Honghui Hospital (Approval Number: 202303051) and was conducted in accordance with the Declaration of Helsinki.

### qRT-PCR

RNA was isolated from 143B cells, TAMs, and human tissues using the UNIQ-10 column RNA Extraction Kit (Sangon Biotech, China). Reverse transcription was performed using the RR047 cDNA Synthesis Kit (TaKaRa, China), and qRT-PCR was performed in a 7500 Real-Time PCR System (Applied Biosystems, Foster City, CA, USA) using 2 × Power SYBR® Green PCR Master Mix (Invitrogen, USA). GAPDH was used to normalize gene expression levels. The following primer sequences were used:

RARRES2-F (5′-CTGATCCCTCTGGCCCTGT-3′), RARRES2-R (5′-TTGGAGAAGGCGAACTGTCC-3′), SOX2-F (5′-CAACCAGAAAAACAGCCCGG-3′), SOX2-R (5′-CGAGCTGGTCATGGAGTTGT-3′), NANOG-F (5′-ACCAGTCCCAAAGGCAAACA-3′), NANOG-R (5′-ACATTAAGGCCTTCCCCAGC-3′), P65-F (5′-GCGAGAGGAGCACAGATACC-3′), P65-R (5′-GCCTGGTCCCGTGAAATACA-3′), P50-F (5′-CCCTACGGAACTGGGCAAAT-3′), P50-R (5′-CCTGGCGGATGATCTCCTTC-3′), IκBα-F (5′-TGCACTTGGCCATCATCCAT-3′), IκBα-R (5′-TCTGTTGACATCAGCCCCAC-3′), IKK-F (5′-ATAAAGGAGATGGGGGCCCT-3′), IKK-R (5′-TTTGATGGGGGATGAAGGGC-3′), GAPDH- F (5′-GCTGCTCTTGGCTCTCAACT-3′), and GAPDH-R (5′-GGCATAGGGCTGGTAATGCT-3′).

### Subcutaneous and orthotopic xenograft tumor models

Nude mice (male, 4 weeks old) were used for the in vivo tumor models. A total of 2 × 10^6^ 143B stable cells were injected subcutaneously or into the cavity of the tibia. Tumor volume was calculated using the following formula: volume (mm^3^) = ab^2^/2. Twenty-eight days after injection, the animals were sacrificed, and the tumors were harvested and fixed in 4% paraformaldehyde. Tumor weight is shown as the mean ± SEM of each group (The Bioethics Committee of the First Affiliated Hospital of Chongqing Medical University (Approval Number: IACUC-CQMU-2023-0436) ).

## Results

### Diversity of cell components in the OS TME and intercellular communication

**Figure 1:**
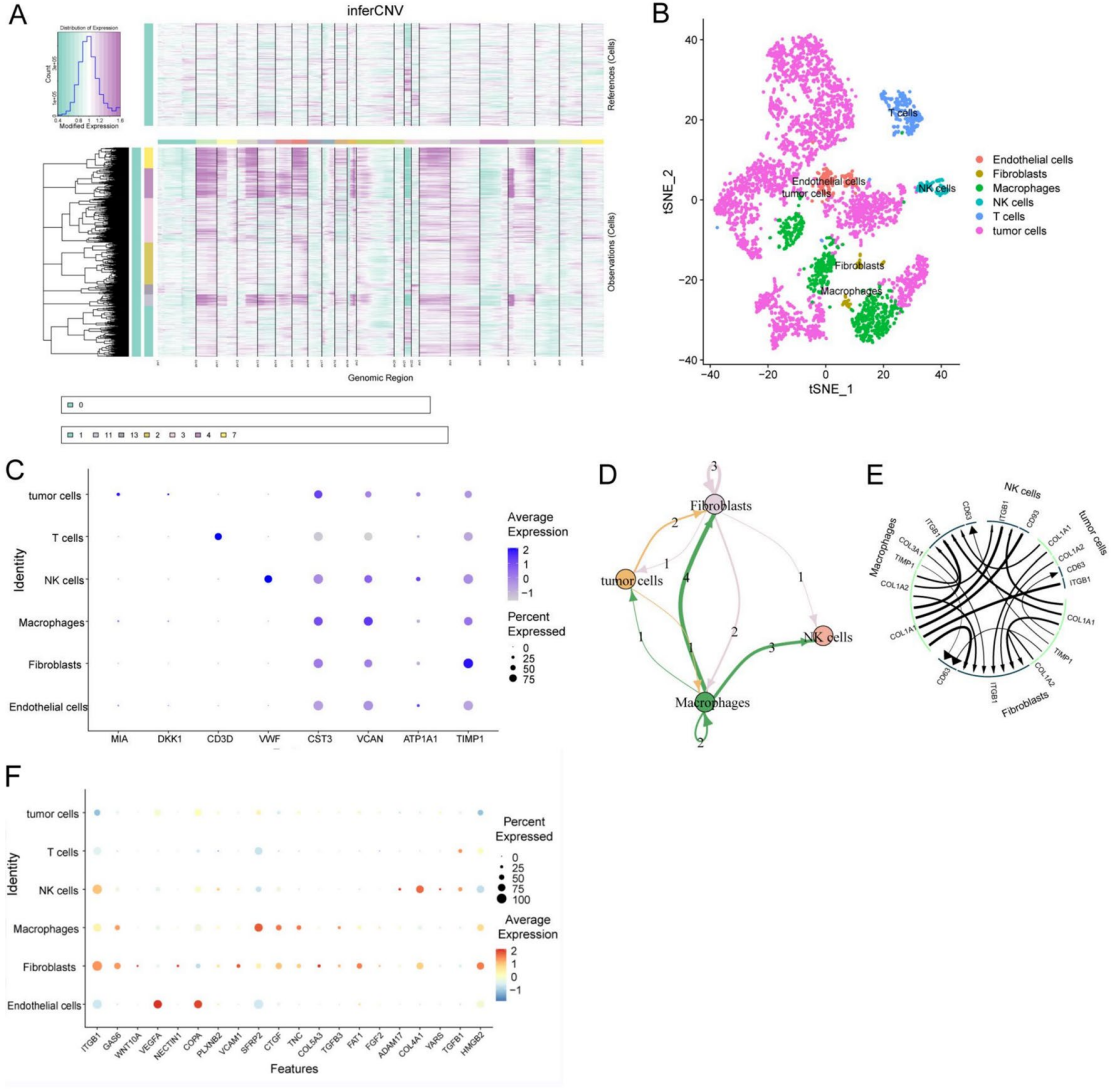
Osteosarcoma TME and intercellular communication analysis (**A**). The hierarchical heatmap shows large-scale copy number variations (CNVs) in lesions from one osteosarcoma sample. (**B**) t-SNE plot showing the six cell clusters, including one malignant cluster from the osteosarcoma sample. (**C**) Dot plots showing the expression of the eight signature genes across the six cellular clusters. The size of the dots indicates the proportion of cells expressing the marker. The color spectrum represents the mean expression levels of the markers. (**D**) Landscape plot of interactions in six cell clusters. The arrow directions show the direction of the cell cluster interactions, where the arrow is the receptor and the nock is the ligand. (**E**) Ligand–receptor interaction plot of the cell clusters. The thickness of the line shows the relative expression levels from high to low. (**F**) Twenty top-ranked ligands used to construct an active ligand–receptor network of cell clusters.

To reveal the landscape of cellular diversity in OS, scRNA-seq analysis of OS single-cell transcriptome data was performed. The cells were clustered into 11 major clusters, and a quality control assessment was carried out (Supplementary File: Supplementary Figs. S1–S6). Next, chromosomal copy number variation (CNV) was calculated by inferCNV to identify malignant cells^28^ (Fig. 1A). After unbiased clustering of cells using t-distributed stochastic neighbor embedding (t-SNE) analyses, six main segregated cell clusters were identified in parallel (Fig. 1B). The cell type of each cluster was identified by singleR according to the expression profiles^29^. The expression of eight signature genes among the cellular clusters, including malignant cells, T cells, TAMs, natural killer cells (NK cells), and cancer-associated fibroblasts (CAFs), was illustrated using dot plots (Fig. 1C). The receptor‒ligand (LR) interaction landscape in the OS microenvironment was subsequently displayed using iTALK analysis. Macrophages, NK cells, and CAFs were highly involved in intercellular interactions with OS cells (Fig. 1D). OS cells interact with TAMs via the COL1A1-ITGB1 signaling pathway, interact with CAFs via the TIMP1-CD63 signaling pathway, and interact with NK cells mainly via the COL1A1-CD93 signaling pathway (Fig. 1E). The results of NicheNet confirmed the results of iTALK by suggesting a crucial role for ITGB1 in the interactions between TAMs and CAFs (Fig. 1F).

### Identification of OSCs and functional diversity of OSC subclusters

**Figure 2:**
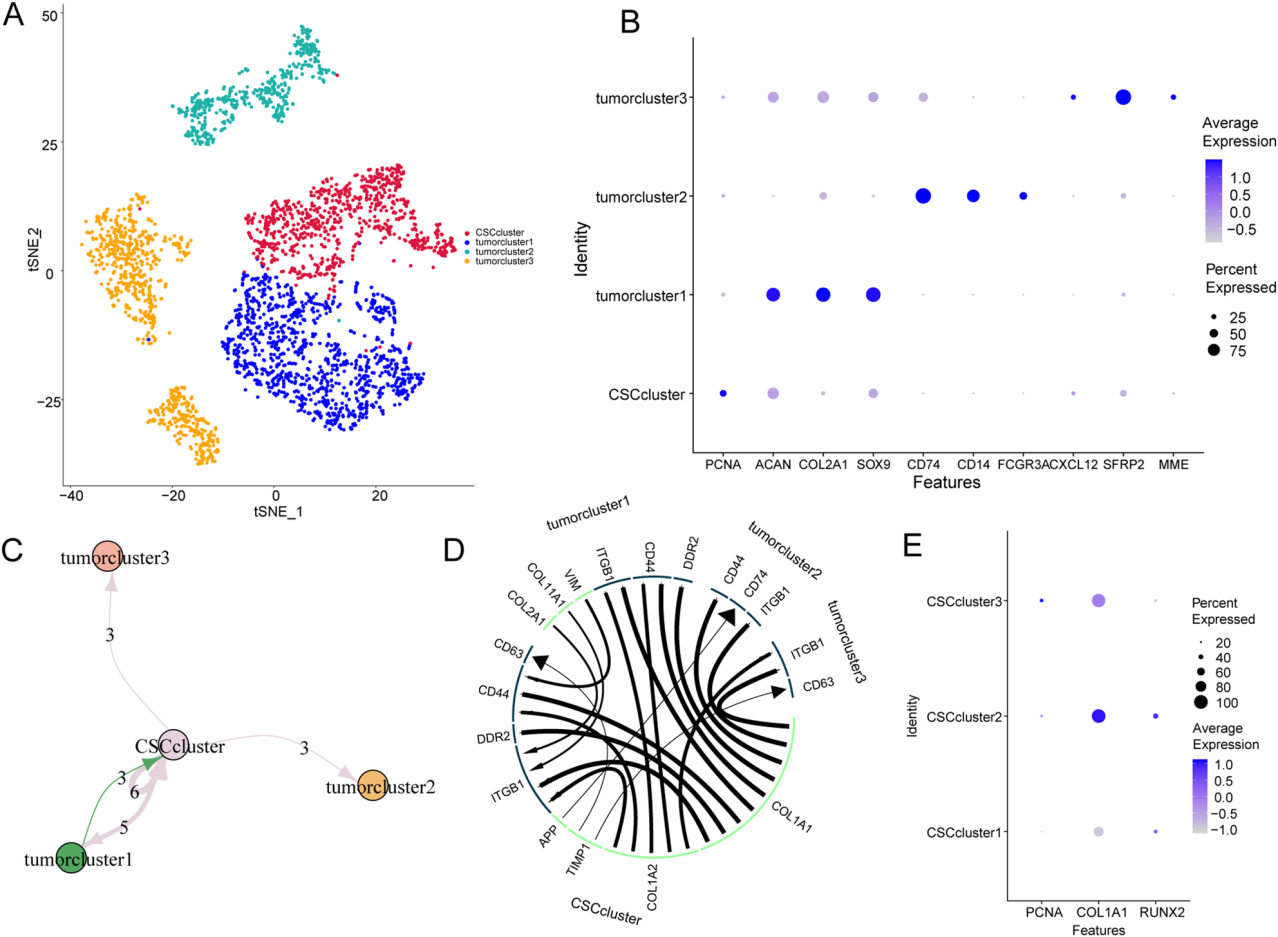
Identification of OSCs. (**A**) t-SNE plot showing the four OS subclusters. (**B**) Dot plots showing the expression of the 10 malignant signature genes across the four OS subclusters. The size of the dots indicates the proportion of cells expressing the marker. The color spectrum represents the mean expression levels of the markers. (**C**,**D**) Ligand–receptor interaction plot for the four OS subclusters. The thickness of the line represents the relative expression levels from high to low. (**E**) Dot plots showing the expression of the three malignant signature genes across the three OSC subclusters.

**Figure 3:**
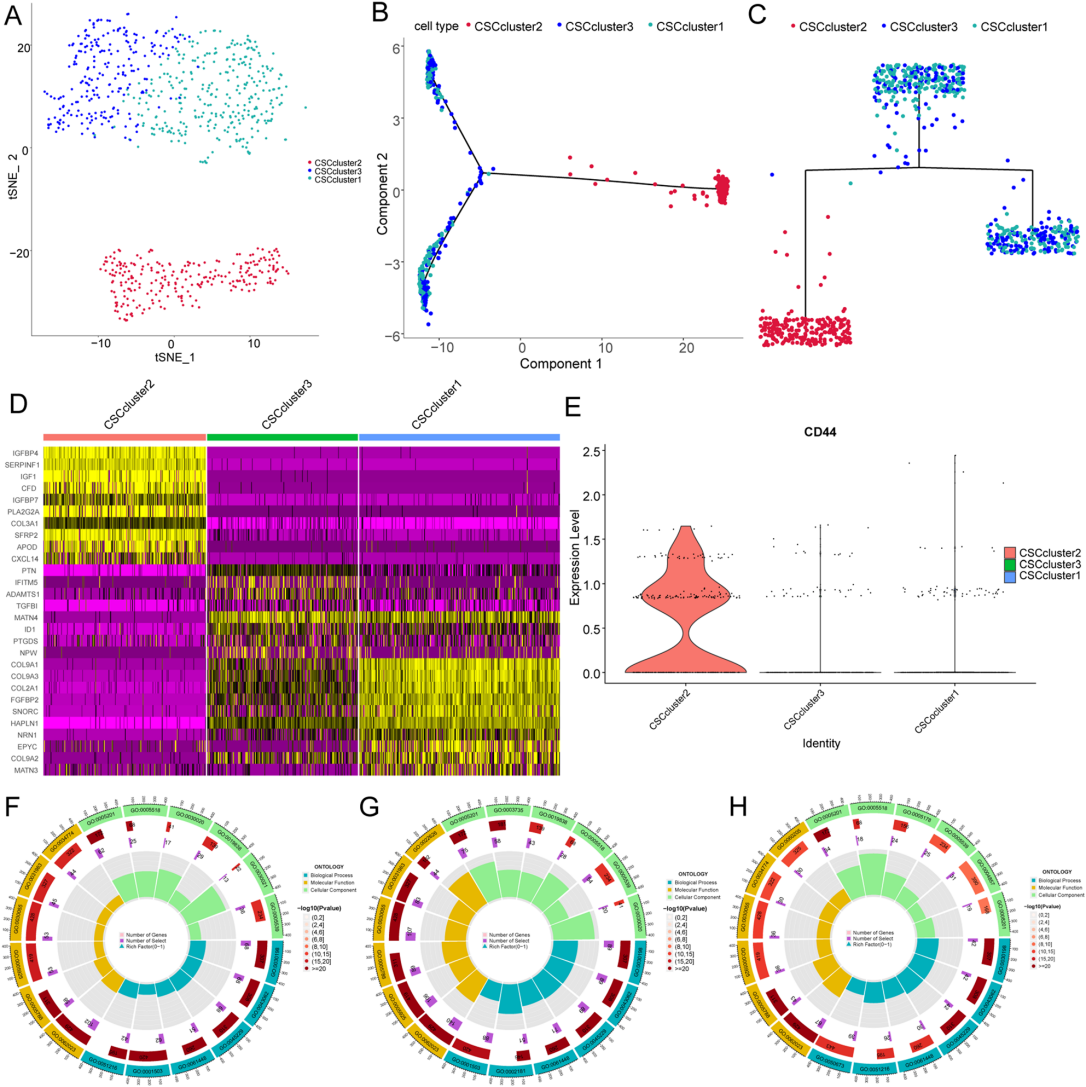
Functional diversity of OSC subclusters. (**A**) t-SNE plot showing the three OSC subclusters. (**B**,**C**) The cell trajectory plot shows the three OSC subclusters. (**D**) Heatmap showing the top DEGs in the OSC subclusters (https://cran.r-project.org/web/packages/pheatmap/index.html). (**E**) Violin plot showing the expression level of CD44 in OSC subclusters. (**F**) Circle plot of enriched GO pathways activities of the OSC Subcluster 1 markers. (**G**) Circle plot of GO pathway activities of the OSC Subcluster 2 markers. (**H**) Circle plot of GO pathway activities of the OSC Subcluster 3 markers.

To further investigate the heterogeneity of OS cells, malignant cells were divided into four subgroups (Fig. 2A). By integrating the results for the expression of cell markers and the results of singleR (Fig. 2B), we found that OS Subgroup 1 expressed high levels of chondroblast markers, such as ACAN, COL2A1, and SOX9, with enriched functions of cytoplasmic translation, ribosome biogenesis, and rRNA metabolic process and thus was defined as a cluster of chondroblast-like OS cells. OS Subgroup 2 expressed high levels of bone marrow cell markers, including CD74, CD14, and FCGR3A; exhibited enrichment of pattern recognition of cytokine production and immune response-regulating signaling pathways; and was defined as a cluster of bone marrow-like OS cells. OS Subgroup 3 expressed high levels of multipotent stem cell (MSC)-related markers, such as CXCL12, SFRP2, and MME (CD10), and exhibited enrichment of the intrinsic components of the Golgi membrane, integral components of the Golgi membrane, the NADH dehydrogenase complex, and the mitochondrial respiratory chain complex; therefore, this group was classified as a cluster of MSC-like OS cells. Interestingly, OS Subgroup 4 expressed high levels of the proliferative osteoblast marker PCNA^30^, which is also known as a cell marker for BM-derived mesenchymal stem cells_._ Moreover, Subgroup 4 appeared at the root in the pseudodifferentiation map (Fig. 2B) and was enriched in the functions of ribonucleoprotein complex biogenesis, osteoblast differentiation, ossification, extracellular matrix organization, and collagen fibril organization. Subgroup 4 was defined as a cluster of OSCs^31^. In addition, analysis of the internal communication between malignant cells revealed that OSCs were mainly ruled as “senders” in the LR interaction relationships (Fig. 2C), and the highly expressed ligands included TIMP1, COL1A2, and COL1A1; the other subclusters functioned as “recipients”. Among these cells, chondroblast-like OS cells had unique pathways including DDR2, whereas bone marrow-like cells had unique pathways including AP-CD74. The LR pathways of MSC-like OS cells included TIMP1-CD63. In addition, feedback was delivered to OSCs by chondroblast-like OS cells via pathways including VIM-CD44 and COL2A1-DDR2 (Fig. 2D). Vimentin (VIM) overexpression is associated with the epithelial–mesenchymal transition process in OS^32^. To further assess heterogeneity within OSCs, we subdivided OSCs to identify the genes and pathways responsible for stemness maintenance, differentiation, and immune regulation. The OSCs were further divided into three subgroups, illustrated as Cluster 1, Cluster 2, and Cluster 3 (Figs. 2E, 3A). The pseudotime differentiation analysis results revealed that Clusters 1 and 3 were more proliferative than Cluster 2 was (Fig. 3B,C). The cell marker expression results revealed an uneven distribution of malignant markers (Fig. 3D,E): Cluster 1 expressed high levels of RUNX2, Cluster 3 expressed high levels of PCNA, and Cluster 2 expressed high levels of COL1A1, indicating the heterogeneity of OSCs. Further functional enrichment revealed that OSCs in Cluster 1 were enriched in the cytosolic ribosome, collagen binding, and collagen-containing extracellular matrix. OSCs in Cluster 2 were enriched in extracellular matrix structural constituents, and OSCs in Cluster 3 were enriched in cartilage development (Fig. 3F–H). By combining the results from these three perspectives, we named Clusters 1, 2, and 3 RUNX2 + proliferative OSCs, COL1A1 + functional OSCs, and PCNA + proliferative OSCs, respectively.

### The communication between different CSC subgroups and TAMs was highly heterogeneous

**Figure 4:**
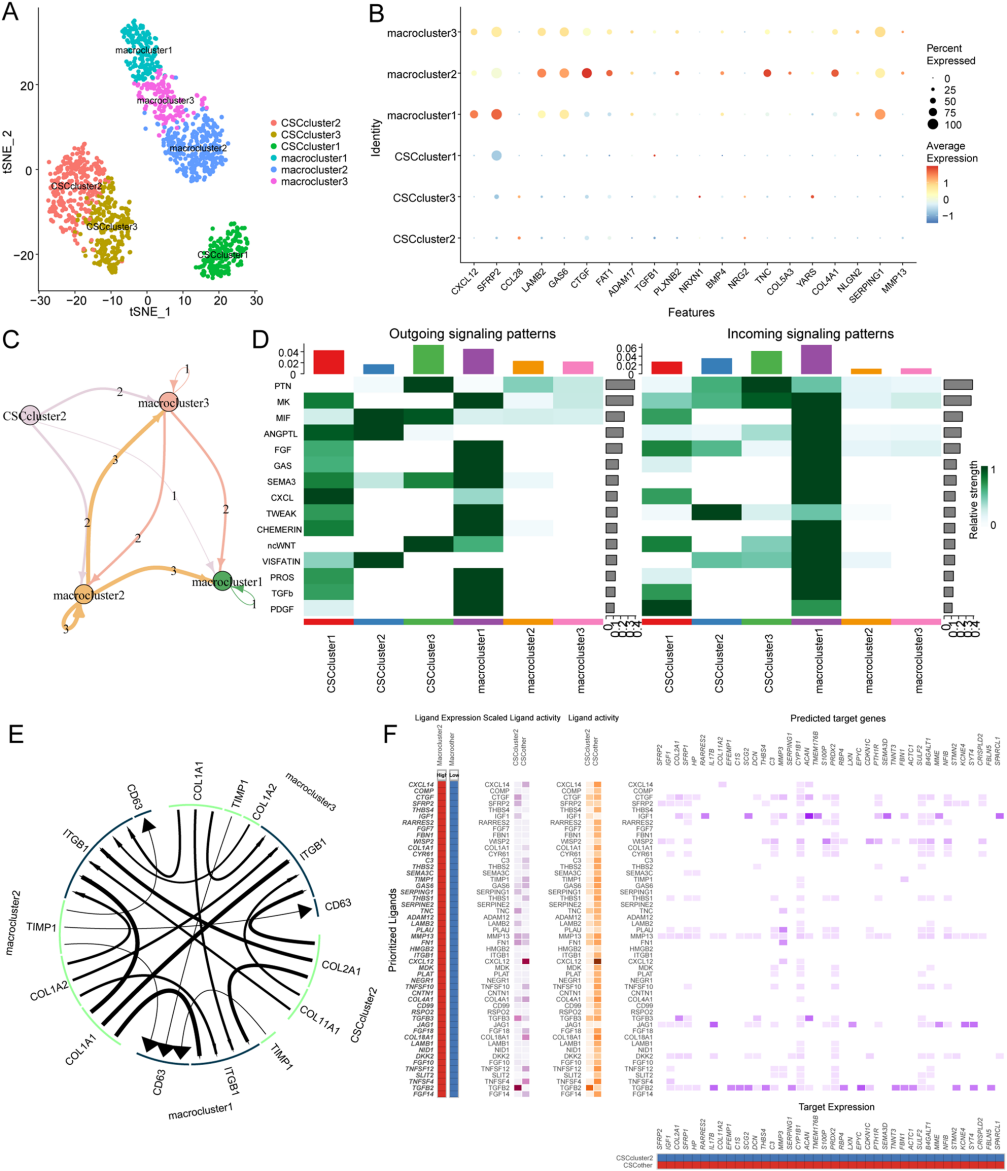
Differential communication analysis between CSC subgroups and TAMs. The t-SNE plot shows the three OSC subclusters and three TAM subclusters. (**A**,**B**) Dot plots showing the ligand–receptor interactions across the three OSC subclusters and three TAM subclusters. The size of the dots indicates the proportion of cells expressing the marker. The colored dots represent the mean expression levels of the markers. (**D**) Heatmap showing the outgoing signaling patterns and incoming signaling patterns of the OSC–TAM communication (https://cran.r-project.org/web/packages/pheatmap/index.html). (**C**,**E**) Ligand–receptor interaction plot for the four OS subclusters. The thickness of the line represents the relative expression levels from high to low. (**F**) Differential communication pattern analysis.

**Figure 5:**
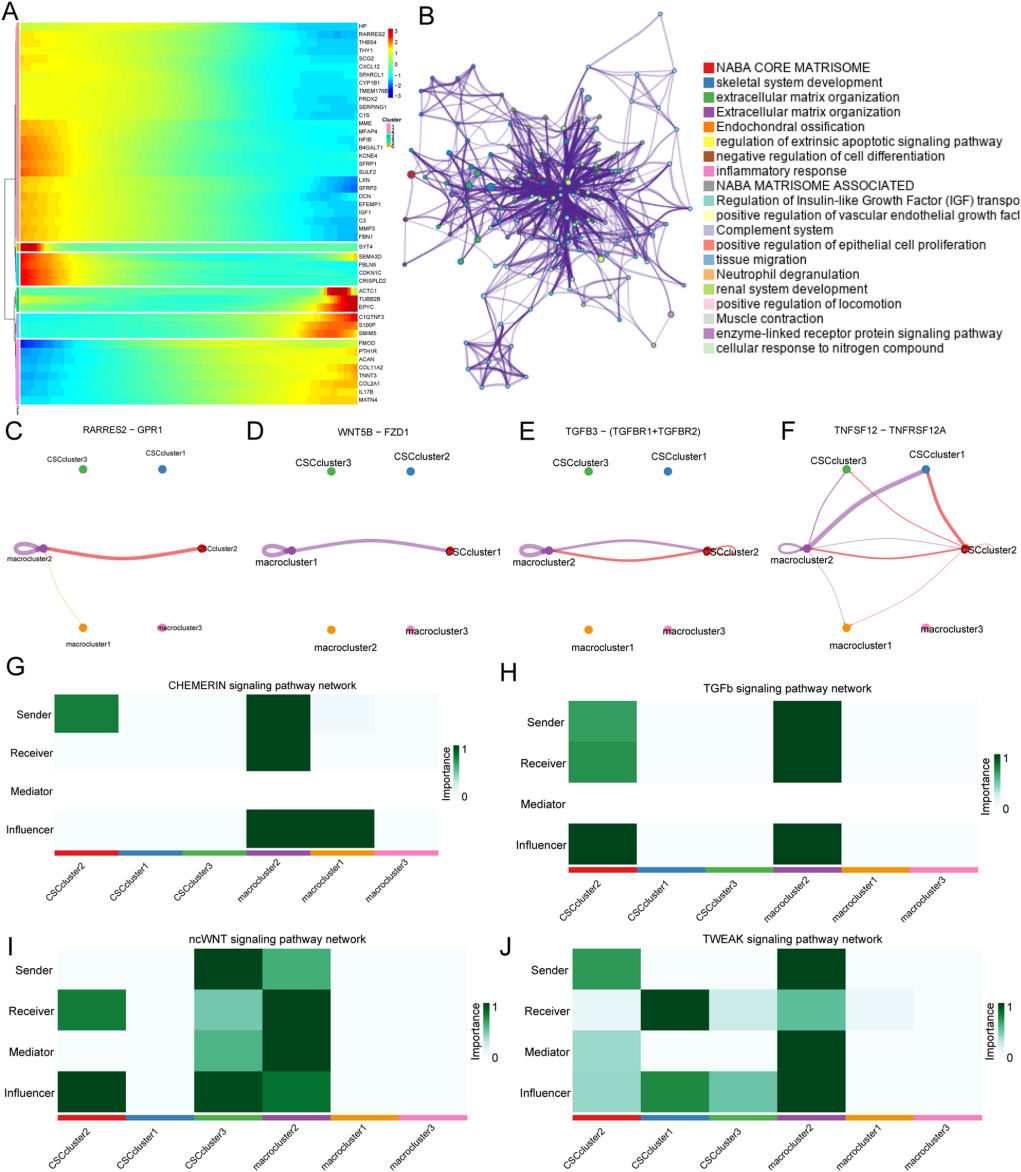
Characterization of OSC–TAM communication-related genes. (**A**) Heatmap showing the expression patterns of OTCRGs according to the pseudotemporal differentiation locus (https://cran.r-project.org/web/packages/pheatmap/index.html). (**B**) GO enrichment analysis of OTCRGs. (**C**–**F**) CircPlot of the cytokine analysis results from the intercellular analysis. (**G**–**J**) Heatmap of the cytokine analysis results from the intercellular analysis.

To investigate the cellular interactions between OSCs and TAMs, we extracted TAMs from Seurat objects and subjected them to subgrouping to observe their phenotypes and functions in OS. TAMs were initially divided into three subgroups: CD68 + TAMs, AHR + TAMs, and M0 TAMs (Supplementary File: Supplementary Figs. S7–S11). To further analyze cell communication between OSCs and TAMs, we extracted and regrouped the cell clusters of OSCs and TAMs (Fig. 4A). The top 20 predicted ligands (Fig. 4B); these included the highly expressed CTGF and GAS6 ligands in CD68 + TAMs with OSCs. In addition, we predicted the specific involvement of CXCL12 and SFRP2 in the crosstalk between AHR + TAMs and COL1A1 + functional stem cells (Fig. 4C). The results of the iTALK analysis showed that COL1A1 + functional stem cells were the key cell subgroup participating in outgoing and incoming communication with TAMs (Fig. 4D), which is consistent with previous predictions. Specifically, the communication pathways between COL1A1 + functional stem cells and CD68 + TAMs, which included COL2A1-ITGB1 and COL11A1-ITGB1, were the most extensive (Fig. 4E). Interestingly, the expression of COL2A1 regulates the differentiation of human mesenchymal stem cells, and its mutation affects cartilage^33^. We further analyzed the receptor–ligand pairs that were differentially expressed between COL1A1 + functional stem cells and CD68 + TAMs compared to the remaining cell groups by applying NicheNet and identified the target genes in COL1A1 + functional stem cells that were significantly regulated by AHR + TAMs (Fig. 4E). We also identified the target genes potentially regulated by OSC–TAM communication (Fig. 4F). We named this set of genes OTCRGs, and the heatmap shows that there are six different expression patterns of the communication genes according to the pseudotemporal differentiation locus (Fig. 5A). Functional enrichment analysis of the OTCRGs revealed the involvement of several biological processes, including the Naba core matrisome, extracellular matrix organization, endochondral ossification, regulation of the extrinsic apoptotic signaling pathway, negative regulation of cell differentiation, inflammatory response, regulation of insulin-like growth factor (IGF) transportation, positive regulation of vascular endothelial growth factor, complement system, positive regulation of epithelial cell proliferation, tissue migration, enzyme-linked receptor protein signaling pathway, and cellular response to nitrogen compounds (Fig. 5B). We also used CellChat to further validate the critical pathways, which included the RARRES2, TWEAK, TGFβ, and WNT pathways (Fig. 5C–F). Our results showed that TWEAK plays a role in signaling in COL1A1 + functional stem cells through an autocrine pathway (Fig. 5G–J). The prediction that TGFβ is involved in the communication between TAMs and OSCs was consistent with the findings of previous research that TAMs promote CSC-like properties via TGFβ-induced EMT and contribute to the progression of hepatocellular carcinoma^33^.

### Communication-related genes characterized different microenvironmental subtypes of OS

**Figure 6:**
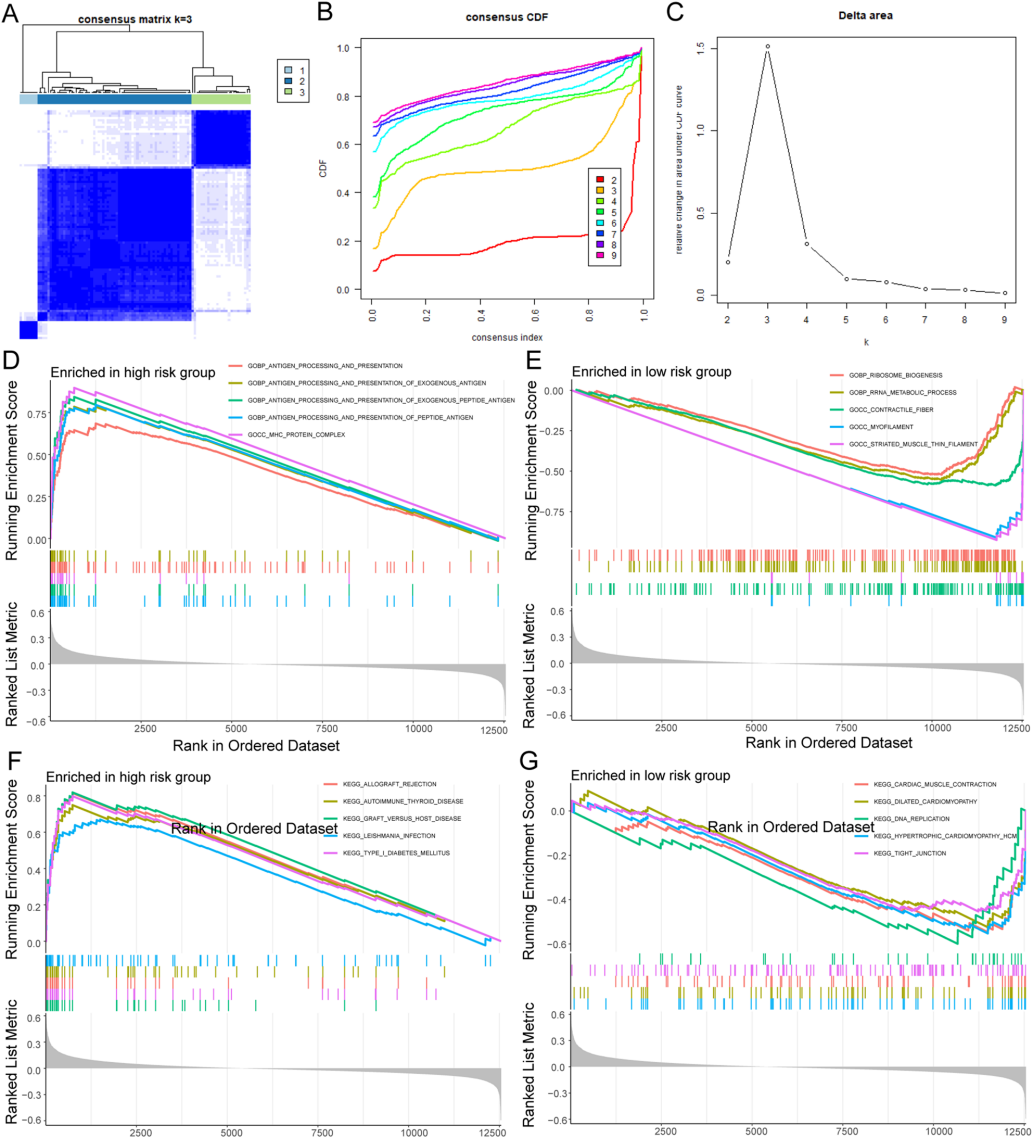
Clustering subtypes of osteosarcoma based on communication-related genes. (**A**–**C**) Consensus clustering identified two distinct clusters of OS with different CTCRG expression patterns. (**D**,**E**). GSVA-KEGG analysis of CTCRG clusters (**F**,**G**) GSVA-GO analysis of CTCRG clusters.

**Figure 7:**
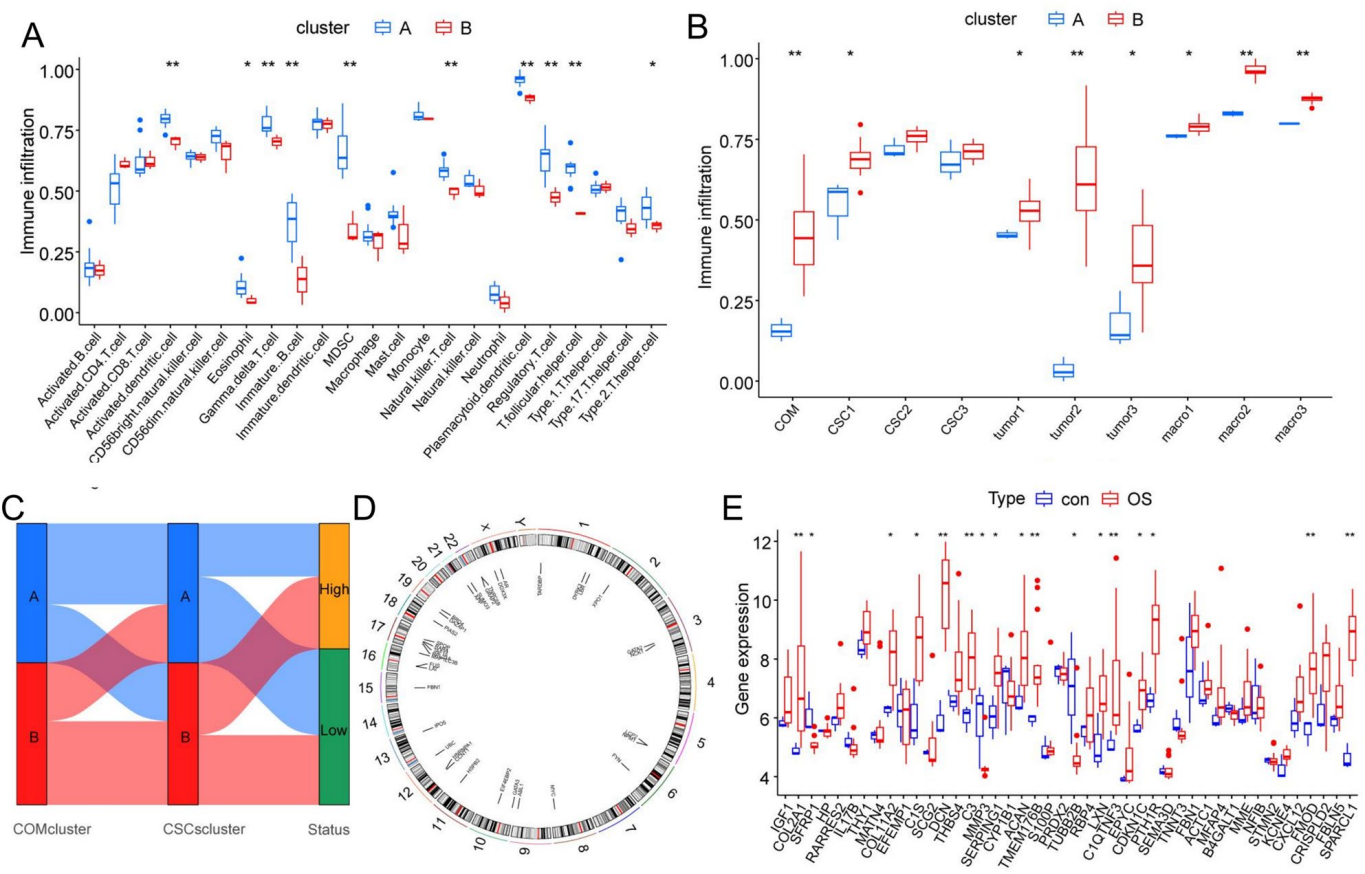
The communication-related subtypes of osteosarcoma exhibit characteristics of different microenvironment profiles. (**A**) ssGSEA and differential analysis of 22 immune cell types. (**B**) ssGSEA and differential analysis of the marker gene sets. (**C**) Sankey diagram showing the repetition degree between the different groups. (**D**) Chromosome locus analysis of genes in OTCRGs. (**E**) Box plot displaying the differences in 44 OTCRGs between the two groups according to the Kruskal–Wallis test; **p < 0.01, *p < 0.05.

To further elucidate the potential role of OTCRGs in the pathology of OS, we first performed an expression analysis of OTCRGs; 17 out of 44 genes exhibited significant differential expression between the OS and control groups (Fig. 7E). Unsupervised clustering based on the ssGSEA scores of the OTCRG gene set was conducted using the ConsensusClusterPlus package to categorize patients with OS into three distinct clusters (Fig. 6A–C); as the sample size of cluster 3 was too small, it was excluded from further analysis. Among the two communication subtypes, differentially expressed genes included those involved in the regulation of autophagy, natural killer cell-mediated cytotoxicity, antigen processing and presentation, endocytosis, lysosome, the RIG I-like receptor signaling pathway, the Toll-like receptor signaling pathway, FC gamma R-mediated phagocytosis, and the B-cell receptor signaling pathway in the GSVA and KEGG analyses (Fig. 6D,E) and positive regulation of oxidative stress-induced cell death, response to type I interferon, antigen processing and presentation of exogenous peptide antigen, antigen processing and presentation of exogenous peptide antigen via MHC class II, antigen processing and presentation of peptide or polysaccharide antigen via MHC class II, negative regulation of the regulated secretory pathway, the Fc receptor signaling pathway, positive regulation of myeloid leukocyte differentiation, and negative regulation of B-cell proliferation in the GO analysis (Fig. 6F,G). To further assess the stemness and TME landscape among the communication subtypes, we applied ssGSEA scores of immune cells and marker gene sets of OSCs and TAM subclusters to compare the TME fractions and OSC scores. As shown in Fig. 7A, among the two stemness clusters, Cluster B exhibited an immunosuppressive subtype characterized by low infiltration of activated dendritic cells, eosinophils, gamma delta T cells, immature B cells, myeloid-derived suppressor cells, natural killer T cells, plasmacytoid dendritic cells, regulatory T cells, and T follicular helper cells; thus, we named this the immune-cold cluster. The enrichment scores of the OTCRGs in each OS sample were quantified by the ssGSEA algorithm, as were the enrichment scores of the cell cluster marker gene sets of the three OSC subclusters and TAM subclusters. Further analysis revealed that Cluster A had a significantly higher percentage of RUNX2 + proliferative OSCs, all subclusters of OS, and all subclusters of TAMs (Fig. 7B). A Sankey diagram confirmed that the communication-related OSCs and communication-related groups exhibited a high degree of overlap (Fig. 7C), and chromosome locus analysis of the genes in the OTCRGs showed that among chromosomes, chromosome 17 contained the largest number of genes in these gene sets (Fig. 7D).

Screening and characterization of OSC–TAM communication-related hub genes.

**Figure 8:**
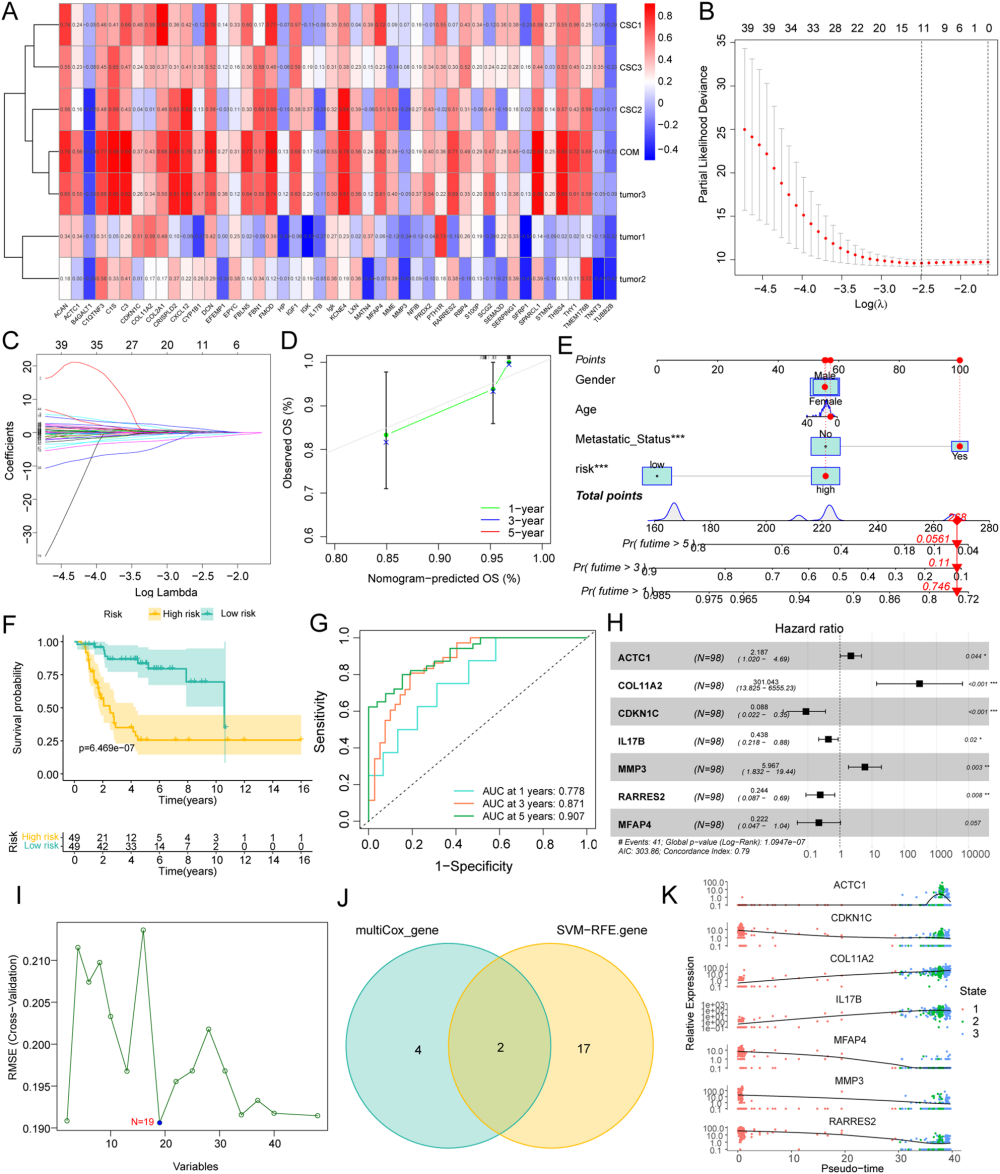
Screening and characterization of OSC–TAM communication-related hub genes. (**A**) Heatmap showing the correlation between OTCRG expression levels and infiltration levels of cell clusters (https://cran.r-project.org/web/packages/pheatmap/index.html). (**B**) Screening of candidate diagnostic genes. The logλ is shown on the horizontal axis, and the cross-validation error is shown on the vertical axis. The cross-validation error is minimal when seven genes are selected. (**C**) The colored lines represent different genes screened by LASSO. (**D**,**F**) Survival analysis of patients according to the CTCRG-based signature. (**E**) Nomogram of the CTCRG-based signature. (**G**) AUC of the CTCRG-based signature. (**H**) Forest plot of the CTCRG-based signature. (**I**) SVM-RFE screening of candidate diagnostic genes. (**J**) The Venn diagram displays the intersection of the results of the two algorithms. (**K**) Pseudotemporal differentiation locus of hub gene expression in OSC clusters.

The expression of OTCRGs in OS was analyzed using the LASSO algorithm (Fig. 8B–C). Among these genes, MFAP4, RARRES2, MMP3, IL17B, CDKN1C, COL11A2, and ACTC1 were first screened. The nomogram and forest plot showed the perfect diagnostic value of this signature (Fig. 8E–H). The area under the curve (AUC) values were 0.778 at 1 year, 0.871 at 3 years, and 0.907 at 5 years (Fig. 8G), and survival analysis indicated the perfect separation potential of the signature to distinguish patients with OS into high- and low-risk groups (Fig. 8D–F). Subsequent single-cell analysis revealed that MFAP4 and RARRES2 were highly expressed in OSC Cluster 1, and MMP3 and IL17B were highly expressed in OSC Clusters 2 and 3 (Fig. 8A). The heatmap showed six different expression patterns of communication genes according to the pseudotemporal differentiation locus analysis; the results indicated that MMP3 was highly expressed at the beginning of differentiation, whereas IL17 was highly expressed at the end of differentiation (Fig. 8K). The OTCRGs were screened using the SVM-RFE algorithm (Fig. 8I). The overlapping genes identified by both algorithms (the hub genes) were RARRES2 and ACTC1 (Fig. 8J). Therefore, these genes were identified as diagnostic genes.

**Figure 9:**
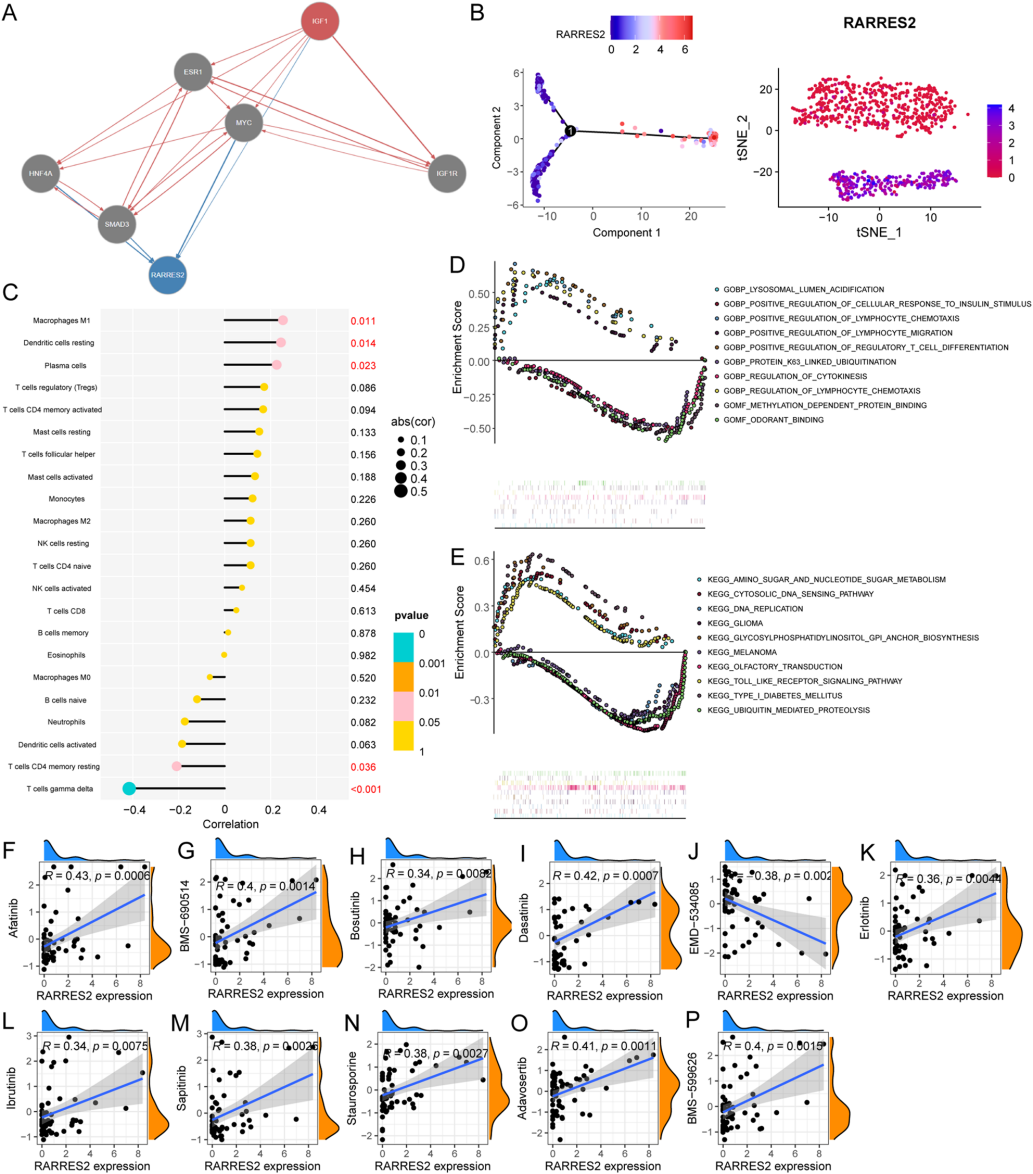
Expression patterns and immune and drug correlations of RARRES2. (**A**) Signaling pathway analysis of the IGF1–*RARRES2* axis. (**B**) Expression pattern of RARRES2 in different clusters of OSCs. (**C**) Correlation analysis between immune cell infiltration and RARRES2 expression in OS. (**D**,**E**) GSEA of the genes associated with the genes whose expression was altered in OS tissues based on the RARRES2-associated DEGs between the high- and low-RARRES2 expression groups. (**F**–**P**) Correlation analysis between drug treatment and RARRES2 expression in OS.

To further investigate the role of *RARRES2* in OS, we first inferred the signaling pathways involved in the interaction between IGF1 and RARRES2 by applying NicheNet, which makes predictions based on the weights of the edges in the integrated ligand-signaling and gene-regulatory networks. Our results indicated that IGF1 affects the regulation of RARRES2 through multiple pathways, including the ESR1, MYC, HNF4A, SMAD3, and IGF1R pathways, which could be crucial in this biological process (Fig. 9A). Next, we examined the expression of *RARRES2* in OSCs at the single-cell level. The highest expression of *RARRES2* was observed in COL1A1 + functional OSCs (Fig. 9B); thus, these clusters of OSCs were renamed RARRES2^high^ OSCs. The association between RARRES2 expression and immune cell infiltration was calculated using Spearman’s correlation analysis (Fig. 9C). The expression levels of RARRES2 were negatively associated with the levels of neutrophils (R =  − 0.313, *p* < 0.001), cytotoxic fine cells (R =  − 0.294, *p* < 0.001), and CD8 T cells (R =  − 0.267, *p* < 0.001) and positively associated with the levels of aDCs (r = 0.177, *p* < 0.001), T helper cells (r = 0.277, *p* < 0.001), and type 2 T helper cells (r = 0.739, *p* < 0.001). The tumor infiltration levels of neutrophils, cytotoxic cells, dendritic cells (DCs), follicular helper T cells (TFH cells), T helper cells, and Th2 cells were consistent with the Spearman’s analysis results. We subsequently performed a differential functional annotation analysis between the high RARRES2 expression group and low RARRES2 expression group by assessing transcriptome data, and GSEA revealed that the high RARRES2 expression group was significantly enriched in various functions, including the GO functions of lysosomal lumen acidification; positive regulation of cellular response to insulin stimulus; positive regulation of lymphocyte chemotaxis; positive regulation of lymphocyte migration; positive regulation of regulatory cell differentiation; K63-linked ubiquitination; regulation of cytokinesis; regulation of lymphocyte chemotaxis; methylation-dependent protein binding (Fig. 9D) and the KEGG functions of amino sugar and nucleotide sugar metabolism, cytosolic DNA-sensing pathway, DNA replication, glioma, glycosylphosphatidylinositol anchor biosynthesis, melanoma, olfactory transduction, Toll-like receptor signaling pathway, type 1 diabetes mellitus, and ubiquitin-mediated proteolysis (Fig. 9E). Next, we performed a drug sensitivity analysis of RARRES2 and screened for drugs with potential effects on RARRES2. RARRES2 expression levels were positively correlated with sensitivity to afatinib, BMS-690514, bosutinib, dasatinib, erlotinib, ibrutinib, sapitinib, staurosporine, adavosertib, and BMS-599626(Fig. 9F–P).

### TAMs promote stemness maintenance in OSCs via the IGF1–*RARRES2* axis

**Figure 10:**
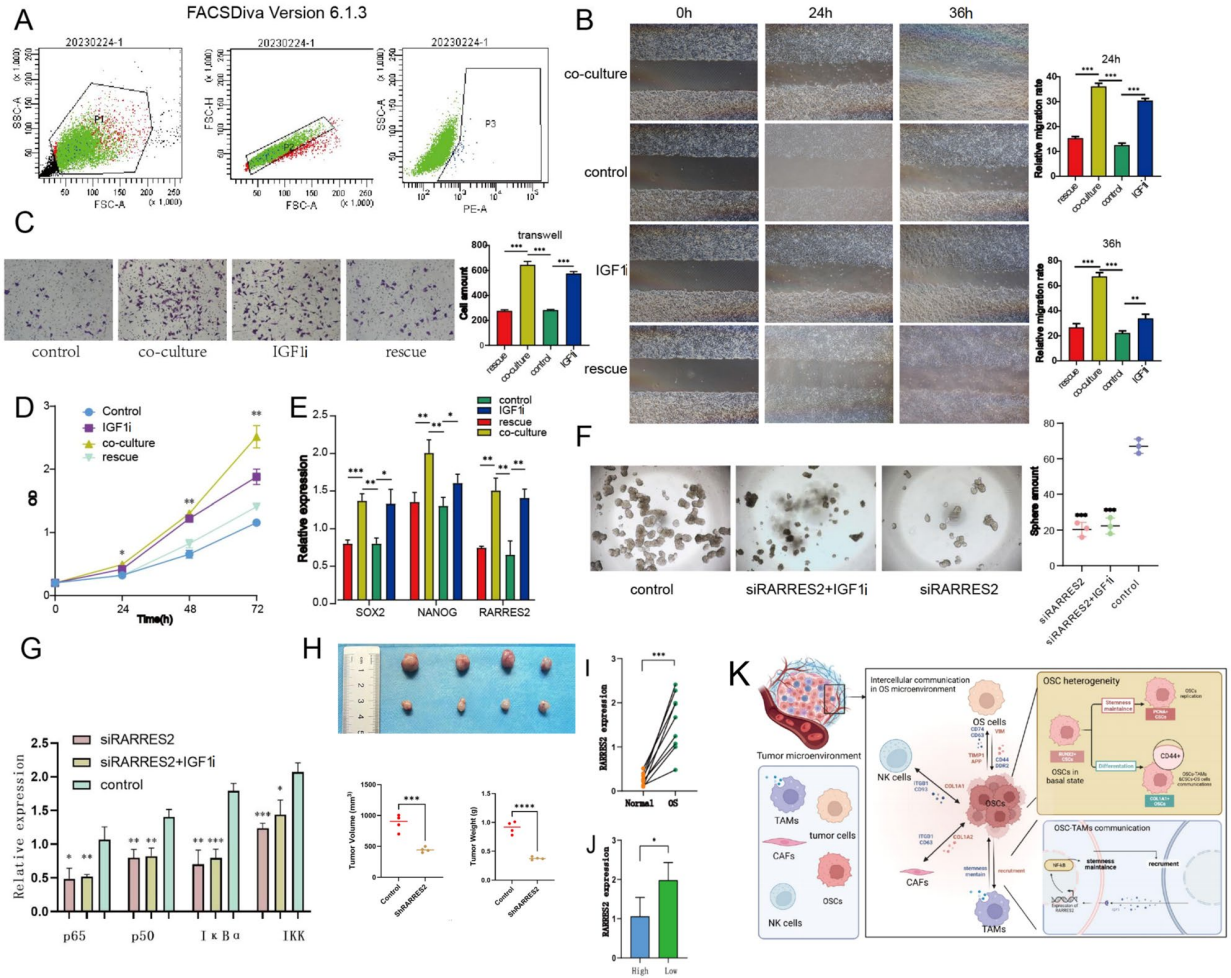
In vitro and patient experiments confirmed the crucial role of the IGF1–RARRES2 axis. (**A**) CD133 immunomagnetic separation miniMACS of 143B cells. (**B**) The results of scratch experiments in different groups. (**C**) The results of transwell experiments in different groups. (**D**) The results of MTT experiments in different groups. (**E**) qRT-PCR results of stemness-related genes in the different groups. (**F**) The results of the sphere formation experiments in the different groups. (**G**) qRT-PCR results of NF-kB pathway-related genes in the different groups. (**H**) tumor models results show shRARRES2 the tumorigenic capacity of osteosarcoma cells was significantly suppressed. (**I**,**J**) qRT-PCR results showing RARRES2 expression in the different groups. All the experimental results were analyzed by the Kruskal–Wallis test, **p < 0.01, *p < 0.05. (**K**) Schematic of the molecular mechanism.

As shown in the Methods section, we first constructed TAM and OSC models. CD133 + cells were first purified using immunomagnetic separation via miniMACS (Fig. 10A). To determine the effect of IGF1i (an IGF1 inhibitor) secreted by TAMs on the phenotypic properties of OSCs, we constructed a TAM-OSC coculture model. The results of MTT, scratch, and Transwell experiments showed that, compared with that of OSCs in the control group, the proliferation, migration, and invasion of OSCs in the TAM coculture and IGF1i groups was promoted; in the rescue group, these behaviors were significantly suppressed after the addition of IGF1 (Fig. 10B–D). In addition, the effects of TAM-secreted IGF1i on the maintenance of OSC stemness were also observed. The qRT-PCR results showed that the expression of stem cell marker genes, including *SOX2* and *NANOG*^34^, was significantly increased in the TAM coculture and IGF1i groups, and RARRES2 expression was also significantly increased (Fig. 10E).

Next, we examined the role of the IGF1–RARRES2 axis in the maintenance of OSC stemness. To evaluate the effect of RARRES2 on sphere formation, siRARRES2 was transfected into OSCs, which were subsequently cultured in a nonadherent culture system containing 50 pg/mL IGF1 (Fig. 10F). The results showed that siRARRES2 cells failed to form spheres regardless of IGF1i treatment. Sphere formation ability was restored by IGF1 treatment in OSCs that were not treated with siRARRES2. To preliminarily explore the mechanism by which RARRES2 maintains OSC stemness, we examined the findings of previous studies on the possible mechanism related to RARRES2 stemness maintenance, and we observed the expression levels of a set of genes related to NF-kB signaling in the cells of each group; these genes have been confirmed to be affected by RARRES2 and are involved in the maintenance of CSC stemness^35^. The results showed that the expression of multiple NF-kB pathway-related genes, including p65, p50, IκBα, and IKK, was significantly downregulated (Fig. 10G). Therefore, RARRES2 may maintain OSC stemness through the NF-kB signaling pathway.Similarly, shRARRES2 was transfected into OSCs, inject into the subcutaneous of nude mice for four weeks, the tumorigenic capacity of osteosarcoma cells was significantly suppressed, leading to a significant reduction in tumor volume and weight (Fig. 10H).

### High expression of RARRES2 in patients with low immune scores

The expression levels of RARRES2 in different patient groups were determined using qRT-PCR. The expression of FLI1 in the tumor tissues (from 23 patients) and samples from the low immunity score group (12 patients) was much greater than that in the adjacent skin tissue (from 23 patients) and the samples of the high immunity score group (11 patients) (Fig. 10IJ).

## Discussion

CSCs reside in unique microenvironments, also known as tumor niches, and their biological behavior is regulated by interactions of these CSCs with immune cells and stromal cells in these niches^36^. However, limited information is available regarding how these CSC populations communicate with other cells in their microenvironments. Targeting the stem cell niche allows the control of CSC genesis and maintenance through cell–cell interactions^37^. Recent advances in single-cell sequencing technologies have enabled routine analyses of intercellular signaling pathways, such as LR pairs, through the coordinated expression of cognate genes according to single-cell gene expression^38^. We provided insights into the subtyping of cells and the communication networks in a multidimensional manner; we also provided insights into the regulatory signaling networks of OSCs and identified a hub axis. In this study, we isolated six main groups of cells, including distinct malignant cells, from OS tissues. The diversity of cell components in the OS TME and in intercellular communication was illustrated. LR interaction results showed that malignant cells extensively interact with immune cells, such as TAMs and NK cells, and with stromal cells, including CAFs. COL1A1-ITGB1 is recognized as a crucial gene in malignant cell communication. Residual chemoresistant, slow-cycling cancer cells (SCCs) regulate CAFs through multiple pathways, including COL1A1-mediated signaling pathways, to establish a growth-promoting TME for SCCs, restoring the proliferative capacity of quiescent non-small cell lung cancer cells. Our results indicated a similar molecular mechanism in OS^39^.

Malignant osteoblasts may originate from any cell type or from the osteogenic differentiation of MSCs. In this study, malignant cells in OS tissues were divided into four main subclusters: chondroblast-like OS cells, marrow-like OS cells, MSC-like OS cells, and OSCs. Genes involved in osteoblast differentiation, ossification, and bone morphogenesis, including COL2A1 and SOX9, were found to be significantly overexpressed in chondroblast-like OS cells. Genes related to RNA methylation and regulation were also significantly overexpressed. Previous studies have shown that chondrocytes can undergo direct transdifferentiation into osteoblasts during endochondral ossification during bone formation^40^. Moreover, the phenotypes of cancer cell subgroups dynamically change during OS^41^. Therefore, we conclude that RNA methylation may contribute to the transdifferentiation of chondroid OS cells into osteoblasts. In addition, compared with OS cells in other subgroups, MSC-like OS cells have higher expression levels of the SFRP2, CXCL12, and MME genes, and overexpression of the SFRP2 gene significantly promotes cell migration and invasion in vitro and enhances metastasis in vivo^42^. CXCL12 is highly expressed in a specific skeletal stromal cell type that coordinates with the BM microenvironment through crosstalk with hematopoietic and endothelial cells and is a candidate cell of origin for at least a subset of primary skeletal tumors^43^. This finding is consistent with our findings, which highlights that intracellular heterogeneity and signaling pathways may drive OS progression and recurrence. CSCs can promote long-term clonal proliferation, tumorigenicity, metastasis, chemotherapy resistance and radiotherapy resistance^44,45^. As the most crucial subgroup in the process of tumor occurrence and development, CSCs play an important role in inducing and maintaining tumor heterogeneity. Recent studies have shown that common types of cancer cells can switch phenotypes to obtain CSC phenotypes under various conditions in the microenvironment^46,47^. Under certain conditions or after the eradication of CSCs, some non-CSCs can be transformed to obtain CSC phenotypes to promote tumor progression^48,49^. OSCs have features similar to those of other CSCs^50^; therefore, we studied the crosstalk between tumor stem cells and tumor cells from the perspective of intercellular communication. The results showed that OSCs were mainly signal senders with high expression of TIMP1, COL1A2, and COL1A1. High expression of TIMP1 promotes stem cell-like traits in tumors by modulating epithelial–mesenchymal plasticity^51,52^; cells with this phenotype act as senders, whereas other tumor cells act as receptors. Among receptor cells, chondroid OS cells exhibit enrichment of unique receptor pathways including the DDR2 pathway. Previous studies have shown that DDR2 is involved in the communication between tumor cells and stromal cells in breast cancer, and its selective extracellular small molecule inhibitor (WRG-2) can delay tumor invasion and migration by inhibiting receptor–ligand interactions^53^. Myeloid OS cells and MSC-like OS cells exhibit enrichment of receptor pathways, including AP-CD74 and TIMP1-CD63 pathways. These findings are consistent with previous findings suggesting that CSCs induce intratumor heterogeneity by generating a cells with different degrees of differentiation, which leads to a range of distinct cell types being present within the tumor^54^. Therefore, tumors are organized hierarchically. Our results also illustrate that chondroblast-like OS cells and OSCs share feedback pathways, including the VIM-CD44 and COL2A1-DDR2 pathways; these findings are also confirmed by other studies that show that the hierarchy induced by CSCs is not a one-way route but is reversible or plastic in the presence of a new hierarchical CSC clone^54^, adding to functional tumor diversity^55,56^. Thus, CSCs have great potential as cancer chemotherapeutic targets. Stem cell-based strategies, particularly those involving specific markers of CSCs, have been used to study CSCs^57^. However, there are currently no specific markers that can accurately represent or identify OSCs in OS tissues or cell lines^46^. This difference may be attributed to the heterogeneity of the OSCs themselves. Thus, we further subgrouped OSCs and showed that OSCs can be divided into RUNX2 + proliferative stem cells, COL1A1 + functional stem cells, and PCNA + proliferative stem cells. The enrichment analysis of OSC subcluster signaling pathways associated with cell marker genes also revealed significant phenotypic differences among these subgroups, and each subgroup was located at different positions during pseudotemporal differentiation, which supported previous theories on the high plasticity of tumor stem cells; that is, the dynamic transformation of phenotypic states occurs through transdifferentiation and reprogramming^58^.

Previous studies have shown that the phenotype and functional changes in CSCs are regulated not only by their own genes but also by the tumor microenvironment. Inflammatory environments provide growth advantages for mutant stem cells. For example, intestinal stem cells with p53 mutations have no competitive advantage over untransformed stem cells; moreover, stem cells are more competitive than their normal neighbors and further promote tumorigenesis^59^. TAMs play a decisive role in shaping the OS microenvironment^60^. In addition, TAMs are involved in crosstalk with CSCs in various types of tumors^61^. M2-type TAMs promote cancer stem-like properties in tumors, including hepatocellular carcinoma^62^, breast cancer^63^, non-small cell lung cancer^64^, pancreatic ductal adenocarcinoma^65^, and glioblastoma multiforme^66^. Mitchum et al. showed that ablation of CCR2 or CSF-1R signaling significantly blocked TAM infiltration in pancreatic ductal adenocarcinoma (PDAC), reduced the number of CD44 + ALDH1 + CSCs, and improved the response to chemotherapy^67^. In addition, ATRA reduces the onset and stemness of OS cells by interfering with M2 TAMs. In our study, we first analyzed the heterogeneity of TAMs in OS tissues based on single-cell data and identified three types of TAMs, CD68 + TAMs, AHR + TAMs, and M0 TAMs, which confirmed that the distribution of M1/M2 macrophage polarization was ambiguous in the actual tumor environment^68^. By further exploring OSC–TAM crosstalk via multiple cell communication analysis methods, we identified the CD68 + TAM subgroup and COL1A1 + functional OSCs as the hub subgroup pair with “lock and key” features. Signaling pathways, including TWEAK, TGFβ, and WNT, are involved in this process. TAMs promote CSC-like properties via TGF-β-induced EMT and may contribute to the investigation of the prognosis of HCC^16^. We subsequently obtained OSC target genes, OTCRGs, which are potentially regulated by TAM–OSC communication, and used them for subsequent studies. First, the OS cohort was divided into two different molecular subtypes using unsupervised clustering according to the OTCRGs. The enrichment of DEGs identified via GSVA in different clusters revealed that multiple natural immunity-related functions, including antigen processing and presentation of exogenous peptide antigens, antigen processing and presentation of exogenous peptide antigens via MHC class II, and antigen processing and presentation of peptides or polysaccharide antigens via MHC class II, were involved. These results confirmed that TAMs and interactions between TAMs and CSCs are crucial in natural tumor immunity^69^. Further ssGSEA also confirmed the considerable difference in immune cell infiltration landscapes between molecular subtypes. The immune-cold phenotype was characterized by low-level infiltration of multiple types of immune cells, including activated dendritic cells, eosinophils, gamma delta T cells, immature B cells, MDSCs, natural killer T cells, plasmacytoid dendritic cells, regulatory T cells, and T follicular helper cells, are involved in shaping the OS TME^70^. We also innovatively applied ssGSEA and single-cell marker gene sets to quantitatively characterize the OSCs and TAM subclusters in each OS cohort. The results showed that compared to samples with other subtypes, samples of the immune-cold subtype had a significantly higher percentage of RUNX2 + proliferative OSCs, all subclusters of OS cells, and all subclusters of TAMs, which confirms our previous results.

Next, to screen for hub genes and their related regulatory molecular mechanisms associated with the lockdown and key features, we first constructed prognostic models related to cell communication by Cox regression, LASSO regression, and SVM analyses, and the hub gene RARRES2 was identified by the intersection of genes contained in different prognostic models. RARRES2, also known as chemerin, encodes a secreted chemotactic protein that has different pro- or antitumor effects on different types of cancer^71–73^. Studies have shown that it can promote the EMT of tumor cells in a variety of tumors to enhance the invasion ability of tumor cells^74^. The chemerin/CMKLR1 axis is one of the main signal transduction pathways involved in tumor progression^74^. To our knowledge, this is the first study to confirm the role of RARRES2 in OSCs and highlight that low expression of RARRES2 leads to decreased tumor aggressiveness. To investigate whether RARRES2 could have a similar effect on other CSCs, we focused on the role of the IGF1–RARRES2 axis in the TAM–CSC interaction by restoring intercellular communication. The chemerin/CMKLR1 axis promotes the interaction between glioblastoma (GBM) cells and TAMs by activating NF-κB signaling^75^. In addition, previous studies have shown that TAMs can promote metastasis and help maintain the stemness of thyroid cancer cells by secreting IGF^76^. Our experimental results indicated that the introduction of TAMs or IGF1 into the culture system promoted the stemness characteristics of OSCs. Based on the above results, we propose the following model: TAM-secreted IGF1 acts on OS cells and upregulates RARRES2 expression, which mediates stemness maintenance through the NF-kB pathway and promotes chemotaxis in TAMs (Fig. 10K).

## Conclusion

OSCs are a highly heterogeneous group of OS cells that are strongly involved in the interaction of multiple types of cells in the OS microenvironment. Notably, TAMs interact with OSCs to induce the maintenance of OSC stemness via the IGF1–*RARRES2* axis.

### Supplementary Information

Supplementary Figure 1.

Supplementary Figure 2.

Supplementary Figure 3.

Supplementary Figure 4.

Supplementary Figure 5.

Supplementary Figure 6.

Supplementary Figure 7.

Supplementary Figure 8.

Supplementary Figure 9.

Supplementary Figure 10.

Supplementary Figure 11.

Supplementary Information 12.

## Data Availability

Restrictions apply to the availability of these data. Single-cell RNA sequencing (scRNA-seq) data were obtained from the Gene Expression Omnibus (GEO) database [https://www.ncbi.nlm.nih.gov/geo/query/acc.cgi?acc=GSE152048]. OS transcriptome sequencing data were downloaded from the Therapeutically Applicable Research to Generate Effective Treatments (TARGETs) database [https://ocg.cancer.gov/programs/target] and the Gene Expression Omnibus (GEO) database (https://www.ncbi.nlm.nih.gov/gds/?term=GSE16088).

## Abbreviations

CNV: Copy number variation
IGF: Insulin-like growth factor
LR: Ligand‒receptor
OS: Osteosarcoma
OSCs: Osteosarcoma stem cells
CSCs: Cancer stem cells
SCC: Slow-cycling cancer cell
TAM: Tumor-associated macrophage
TME: Tumor microenvironment
OTCRGs: OSC–TAM communication-related genes
ROC: Receiver operating characteristic
BM: Bone marrow
NK cells: Natural killer cells
CAFs: Cancer-associated fibroblasts
t-SNE: T-Distributed stochastic neighbor embedding
DC: Dendritic cell
TFH cells: Follicular helper T cells
