# Supplementary figures and images for: RARRES2 is involved in the “lock-and-key” interactions between osteosarcoma stem cells and tumor-associated macrophages
Source: Sci Rep. 2024 Jan 27;14:2267. doi: 10.1038/s41598-024-52738-5 (PMC10821905; doi:10.1038/s41598-024-52738-5)

**-0.22**

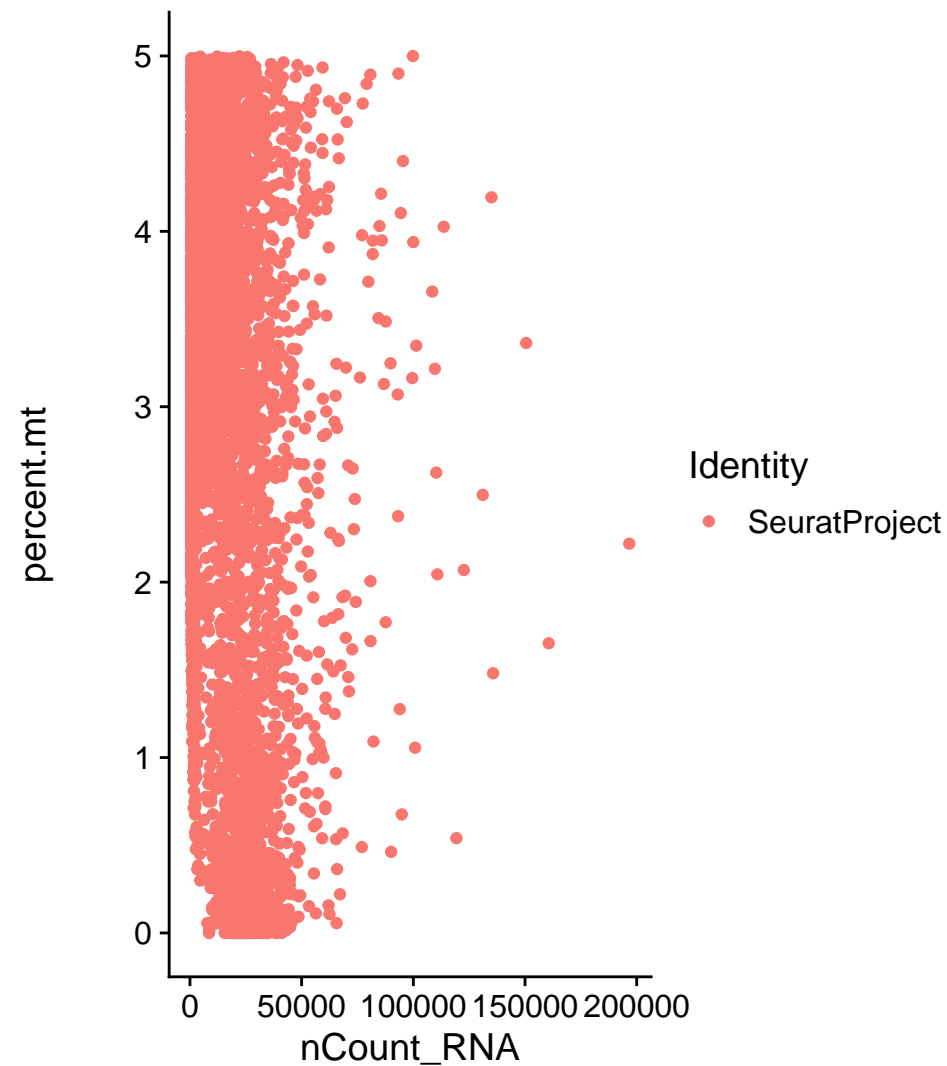

**0.89**

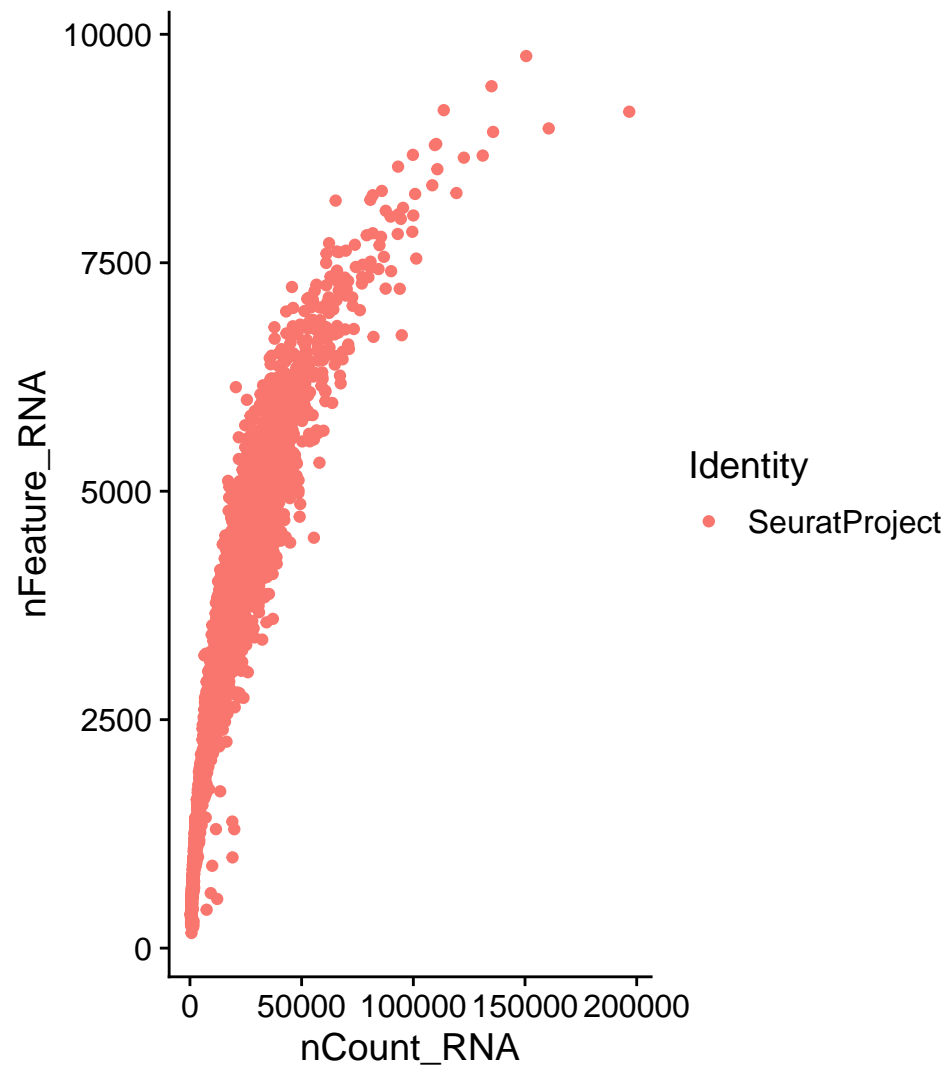

Supplement: Supplementary file 1 — Supplementary Figure 1. [file 41598_2024_52738_MOESM1_ESM.pdf]

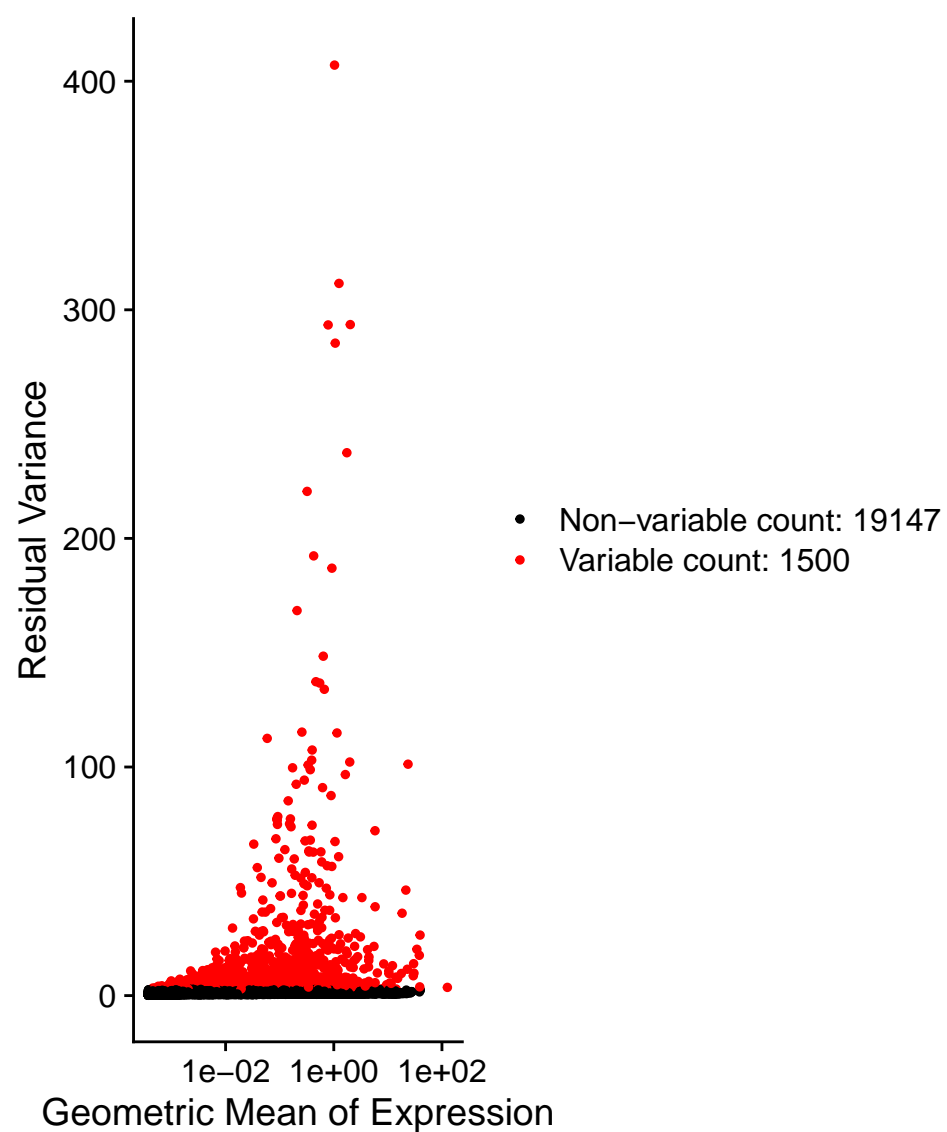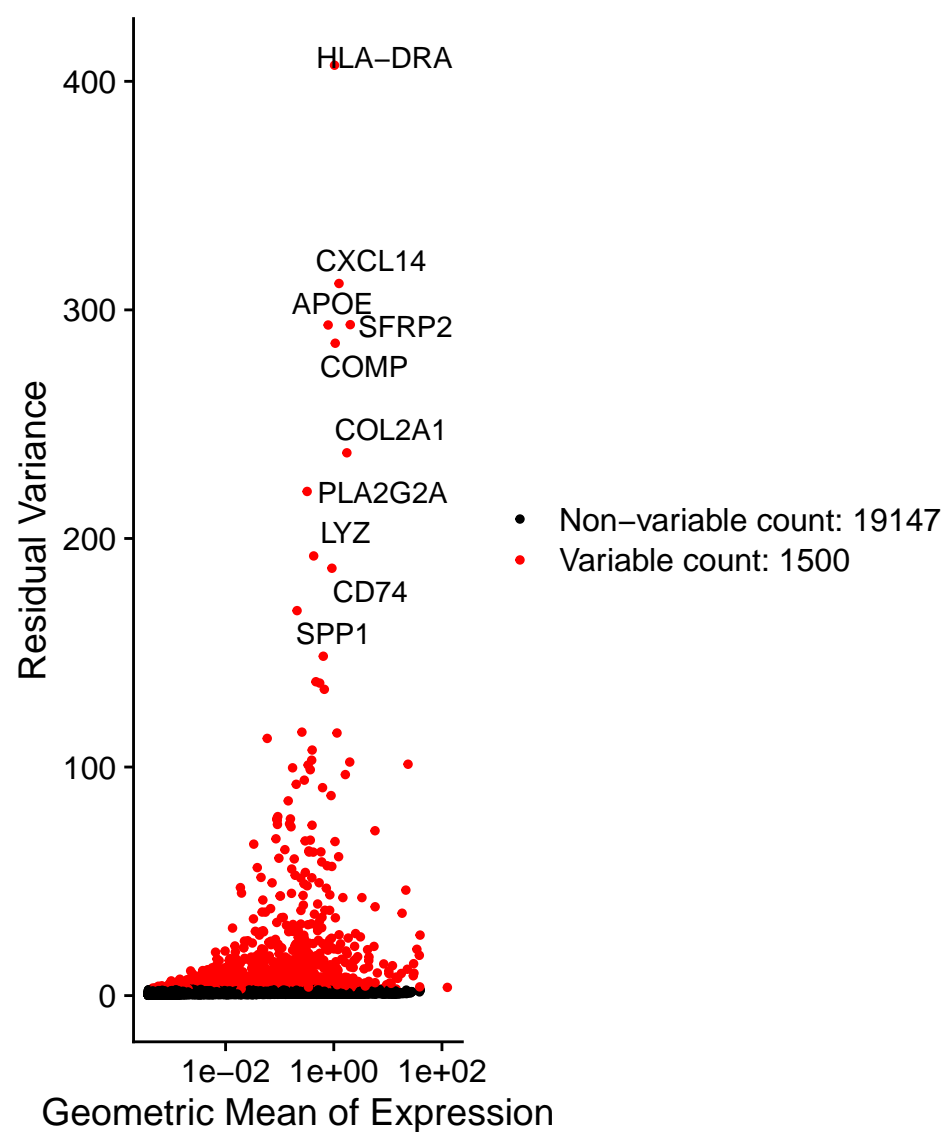

Supplement: Supplementary file 2 — Supplementary Figure 2. [file 41598_2024_52738_MOESM2_ESM.pdf]

**nFeature\_RNA**

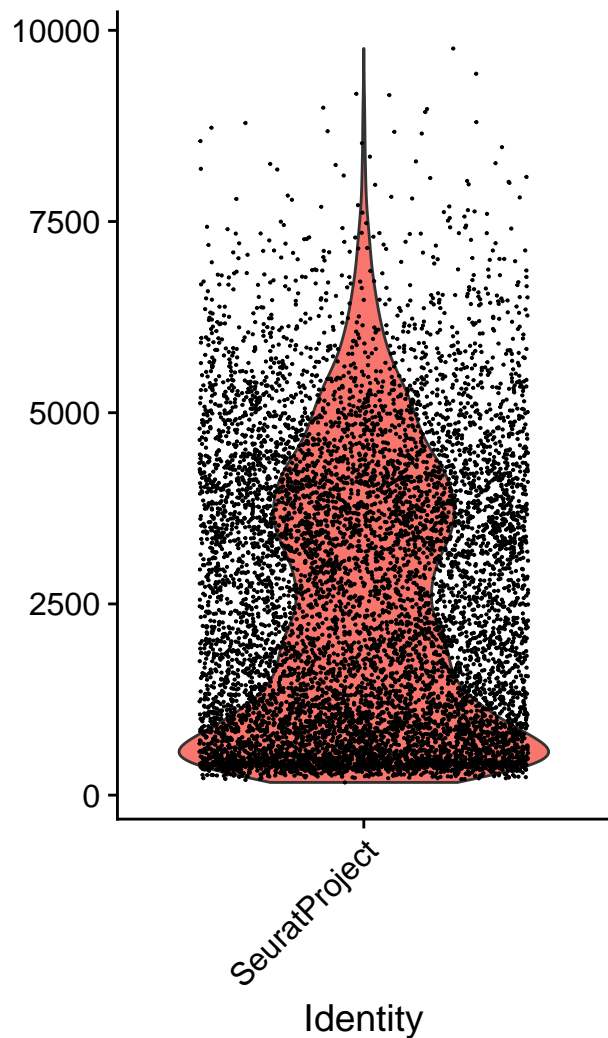

**nCount\_RNA**

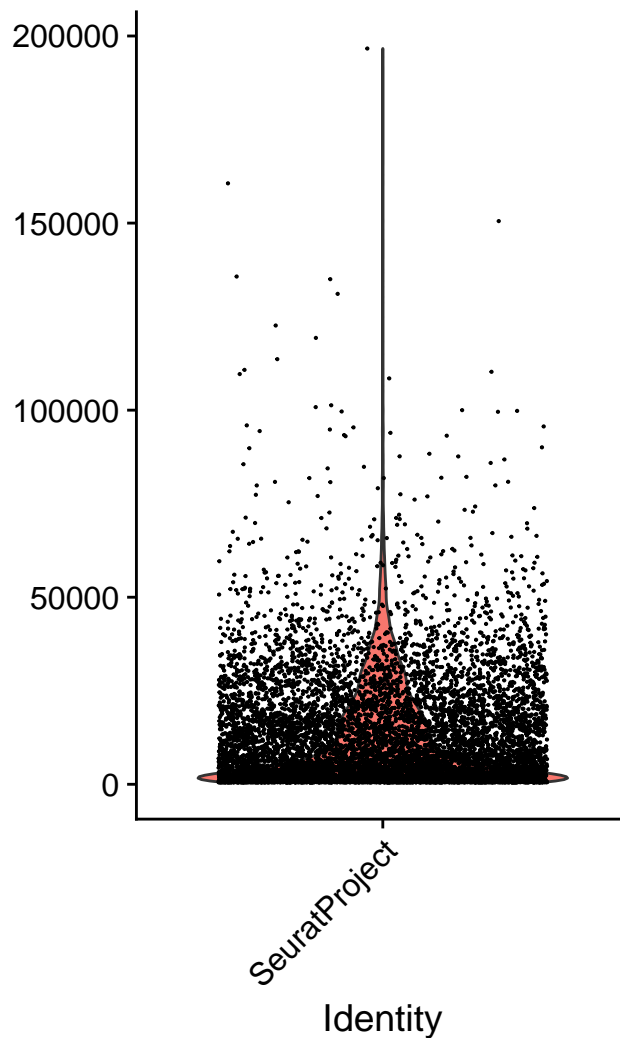

**percent.mt**

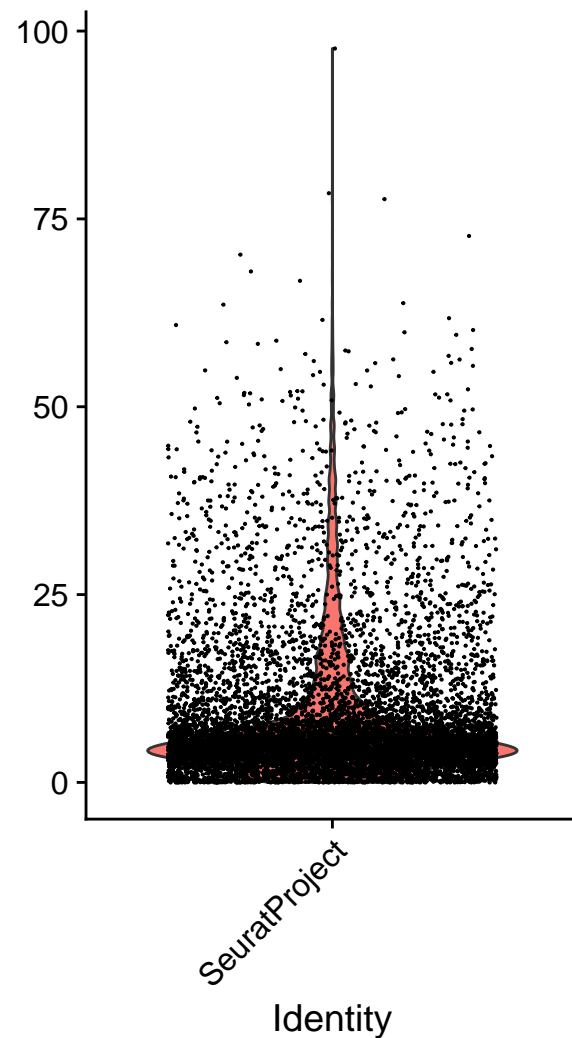

Supplement: Supplementary file 3 — Supplementary Figure 3. [file 41598_2024_52738_MOESM3_ESM.pdf]

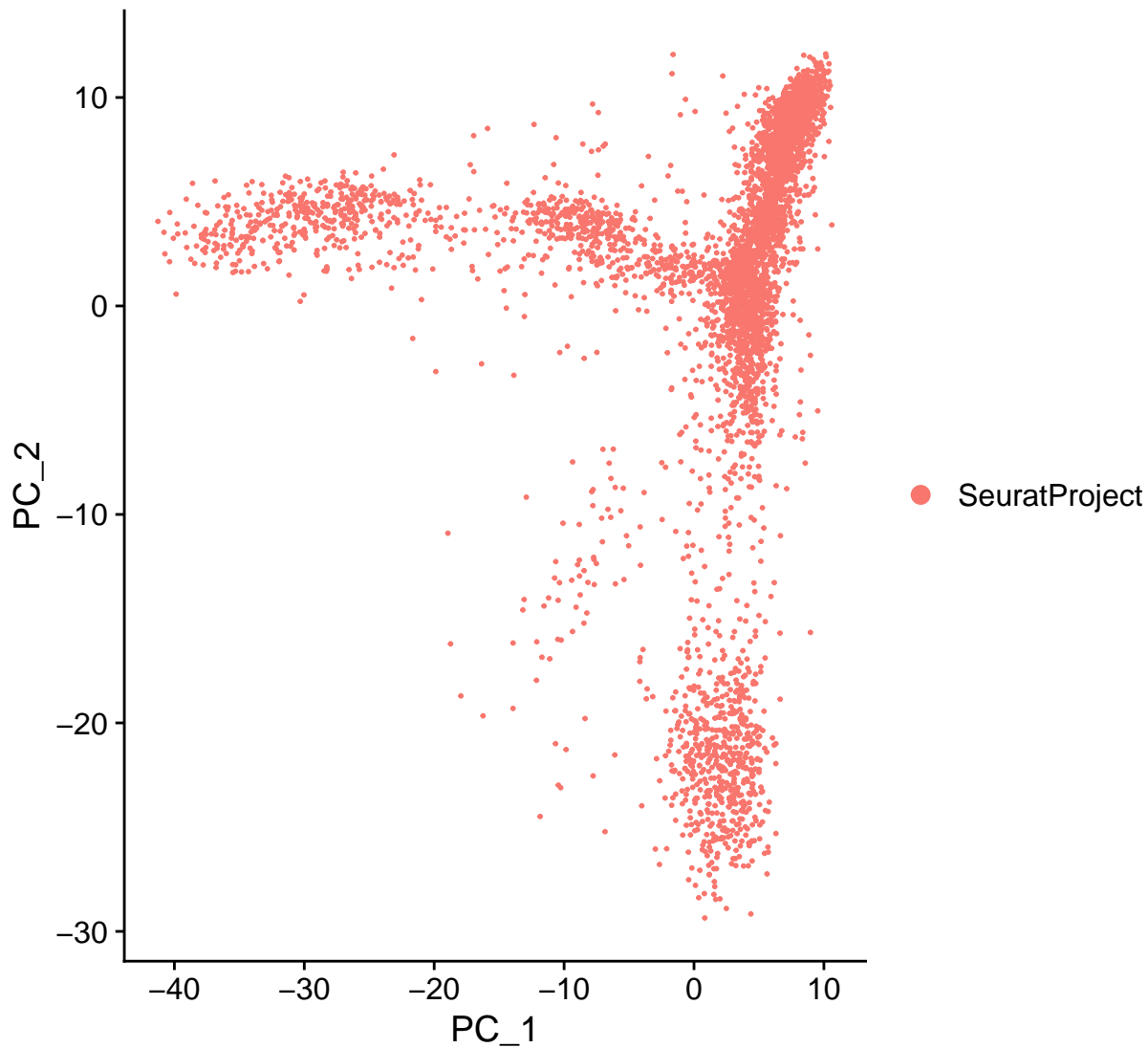

Supplement: Supplementary file 4 — Supplementary Figure 4. [file 41598_2024_52738_MOESM4_ESM.pdf]

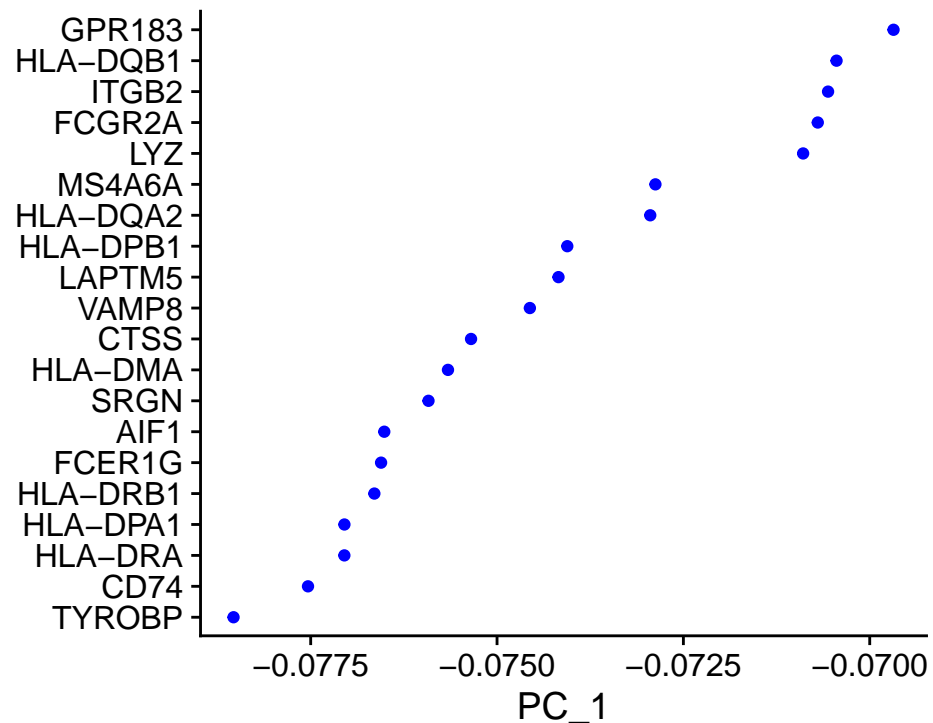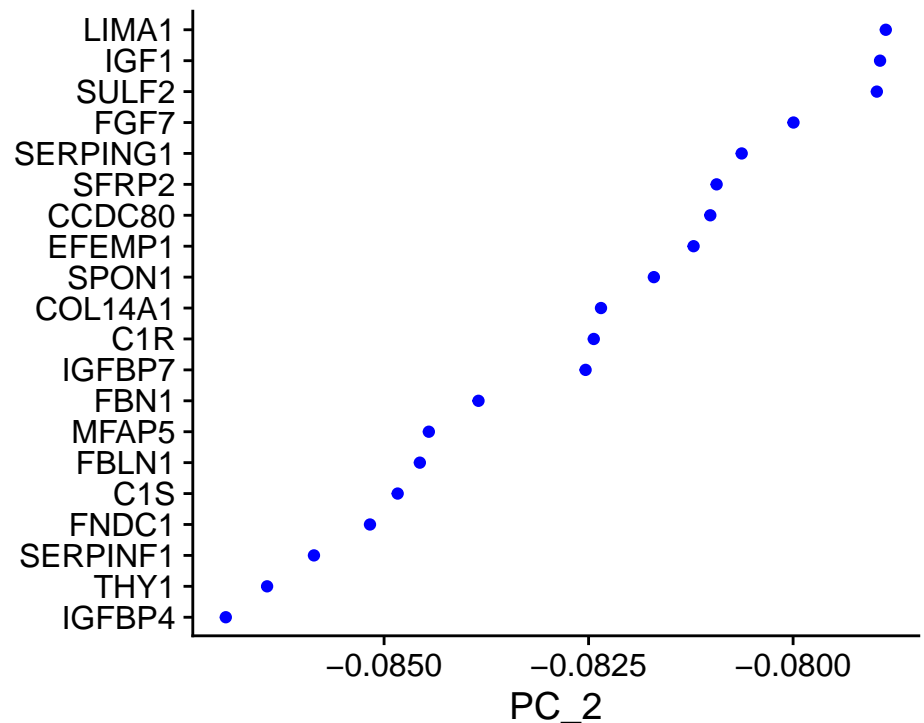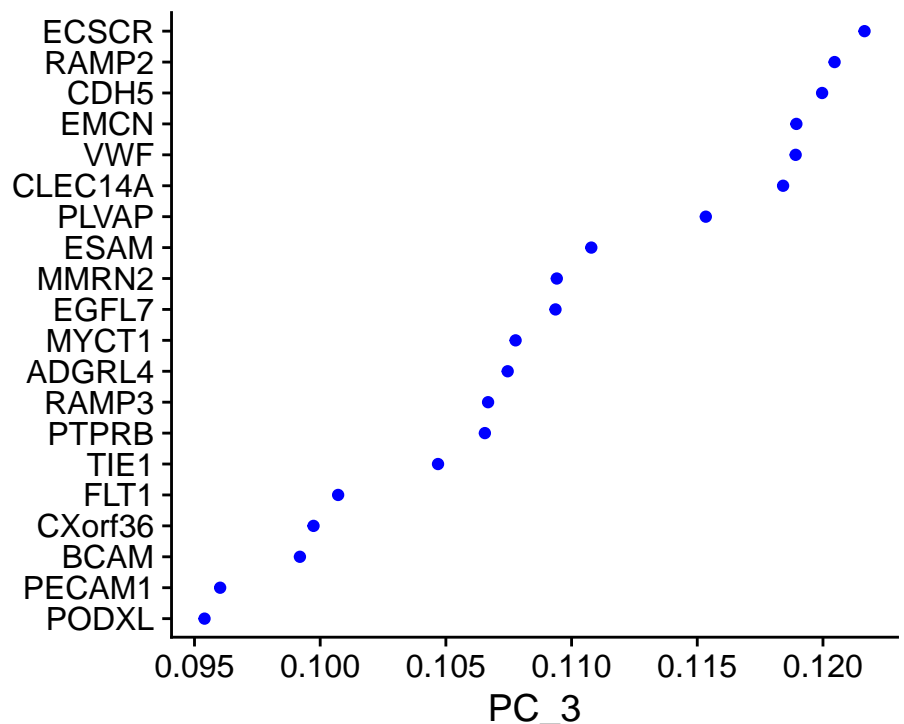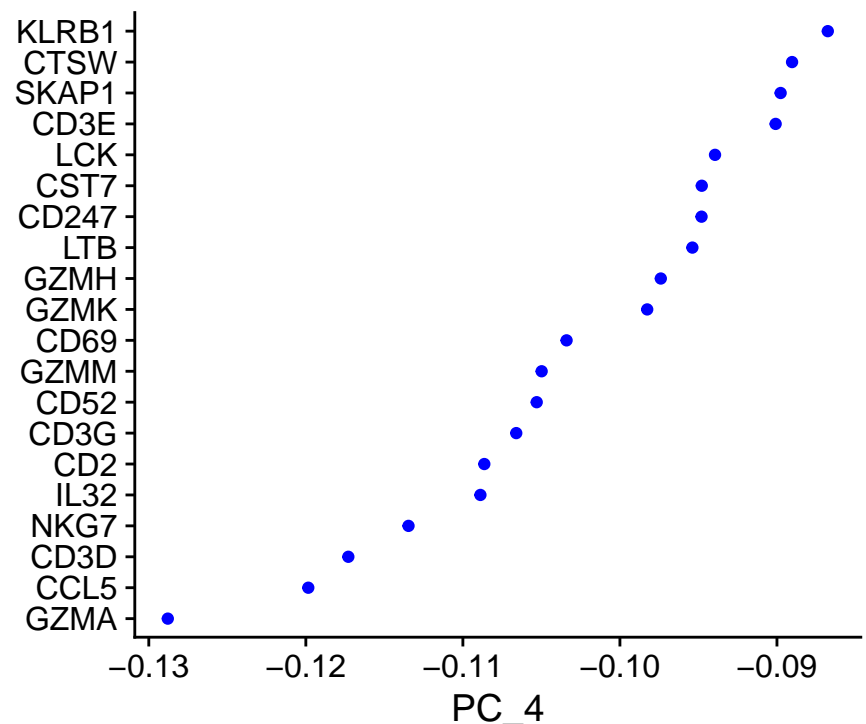

Supplement: Supplementary file 5 — Supplementary Figure 5. [file 41598_2024_52738_MOESM5_ESM.pdf]

PC\_1

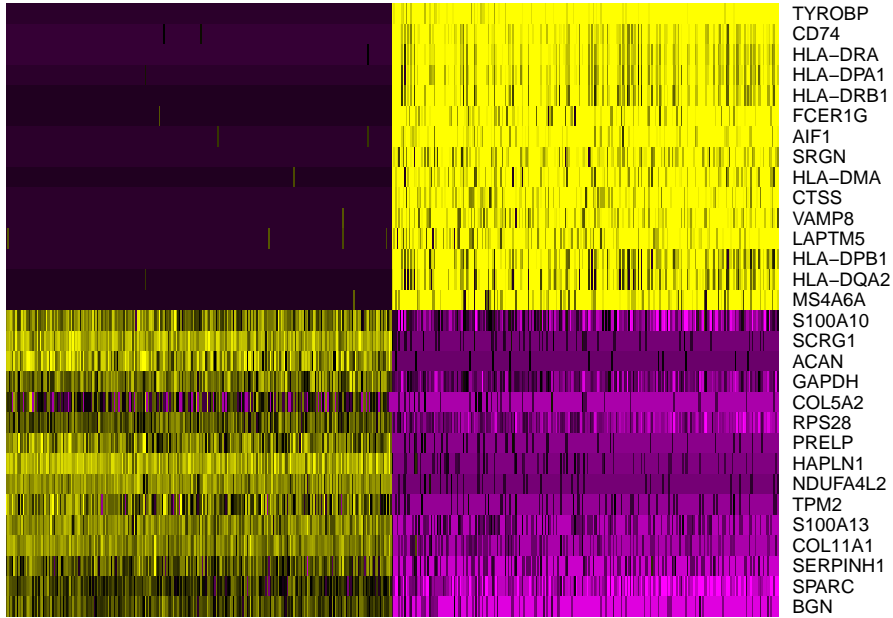

PC\_2

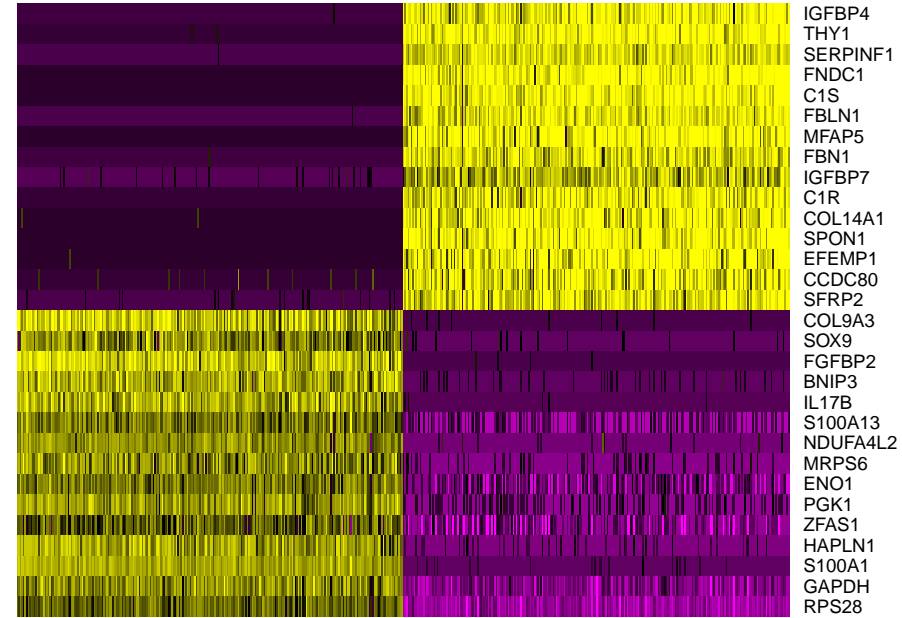

PC\_3

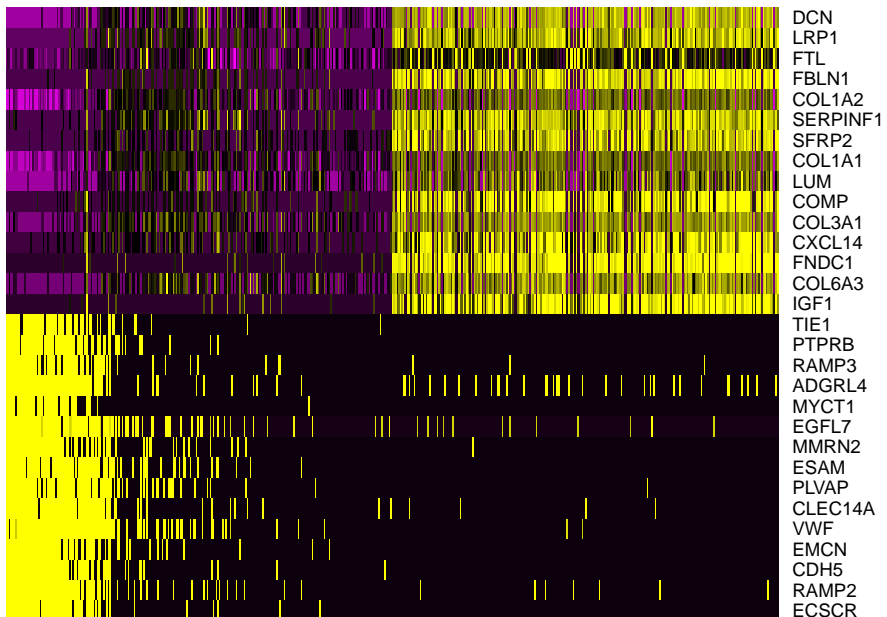

PC\_4

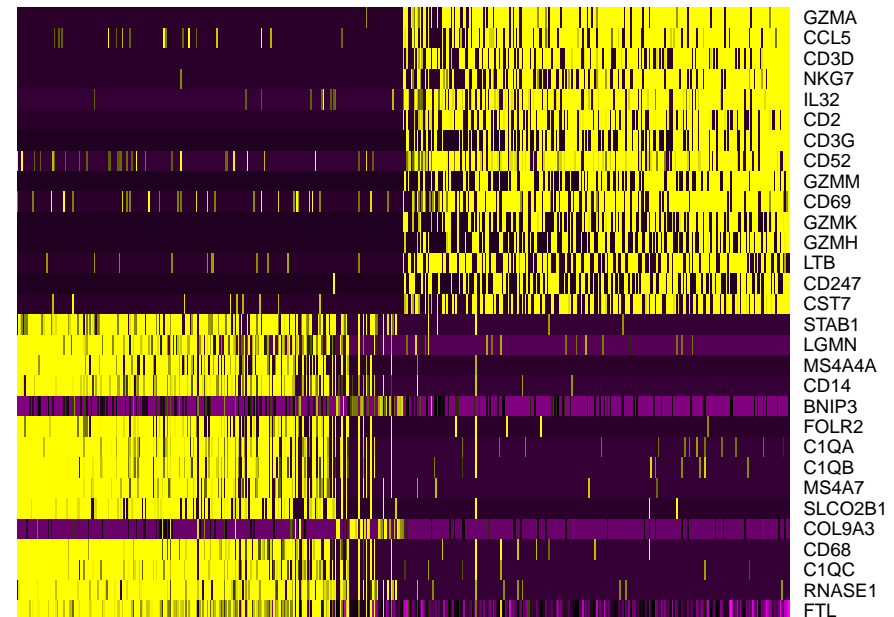

Supplement: Supplementary file 6 — Supplementary Figure 6. [file 41598_2024_52738_MOESM6_ESM.pdf]

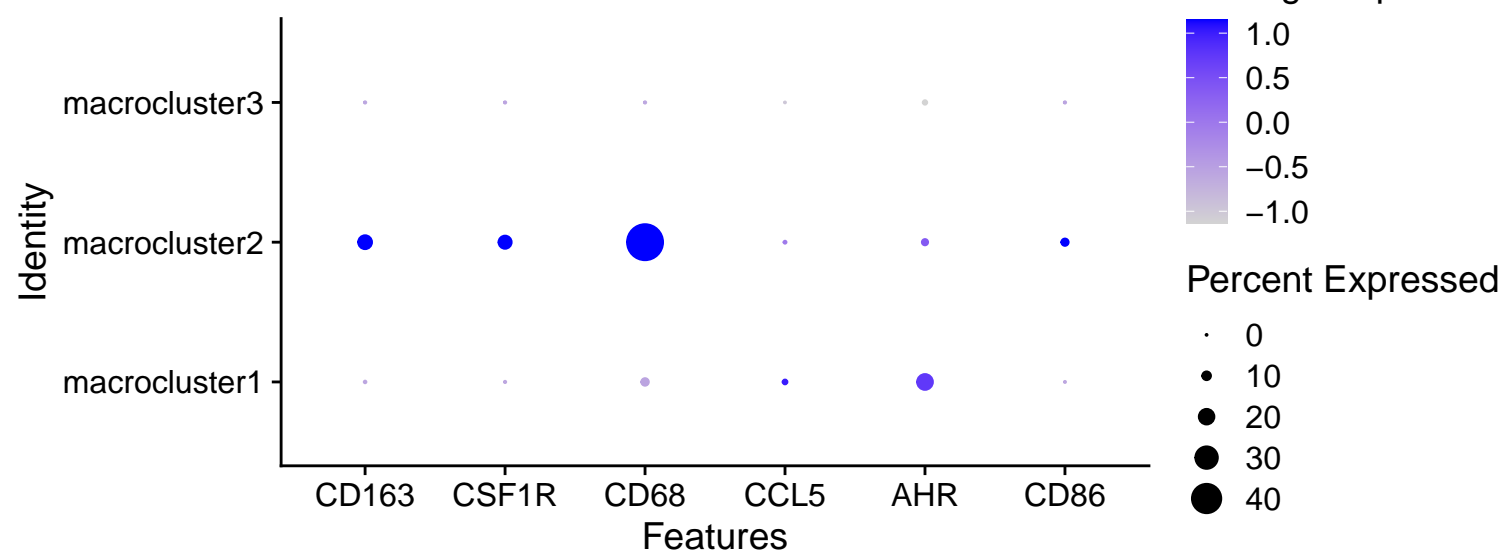

Supplement: Supplementary file 7 — Supplementary Figure 7. [file 41598_2024_52738_MOESM7_ESM.pdf]

cell\_type2    clustermacrocluster1    clustermacrocluster2    clustermacrocluster3

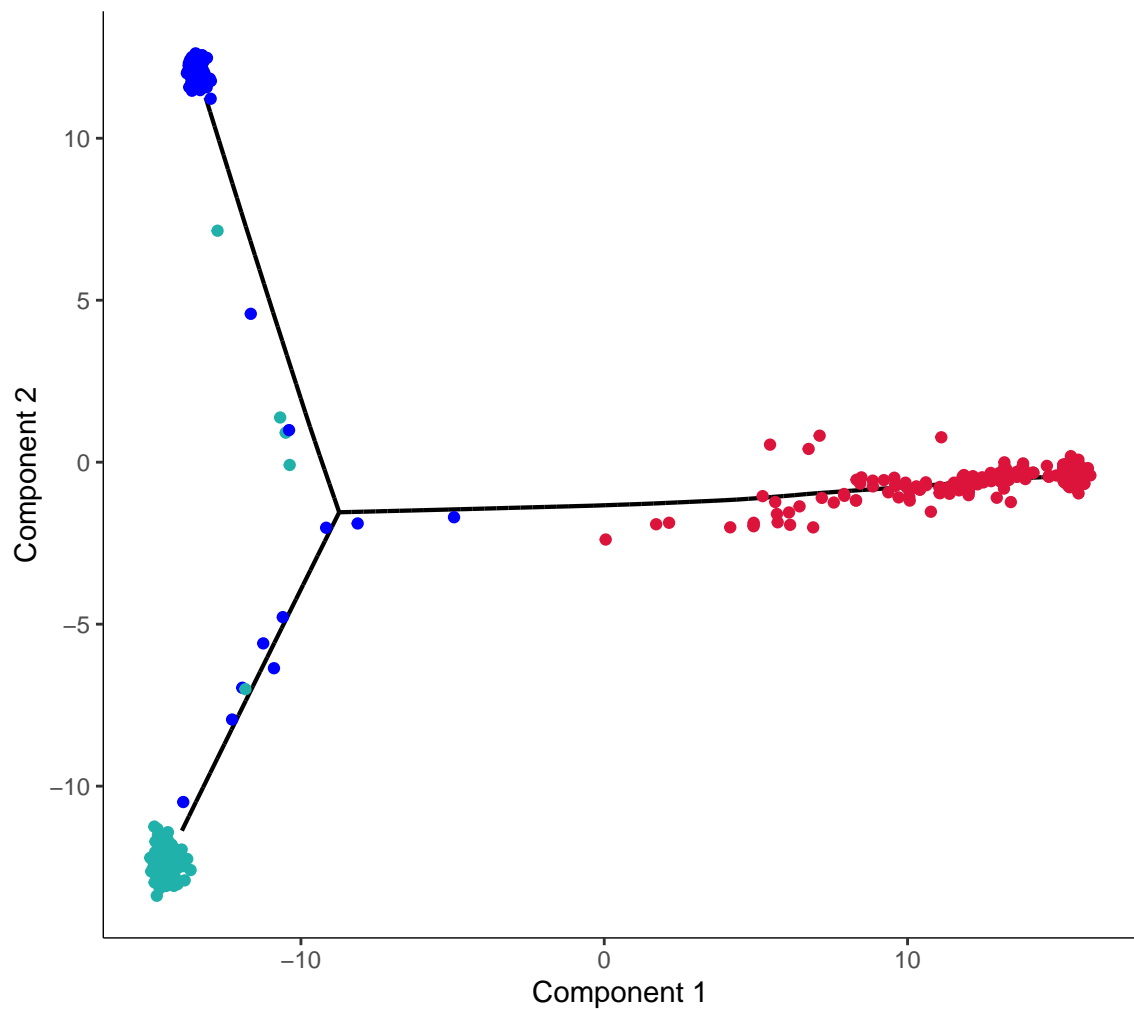

Supplement: Supplementary file 8 — Supplementary Figure 8. [file 41598_2024_52738_MOESM8_ESM.pdf]

cell\_type2    ● clustermacrocluster1    ● clustermacrocluster2    ● clustermacrocluster3

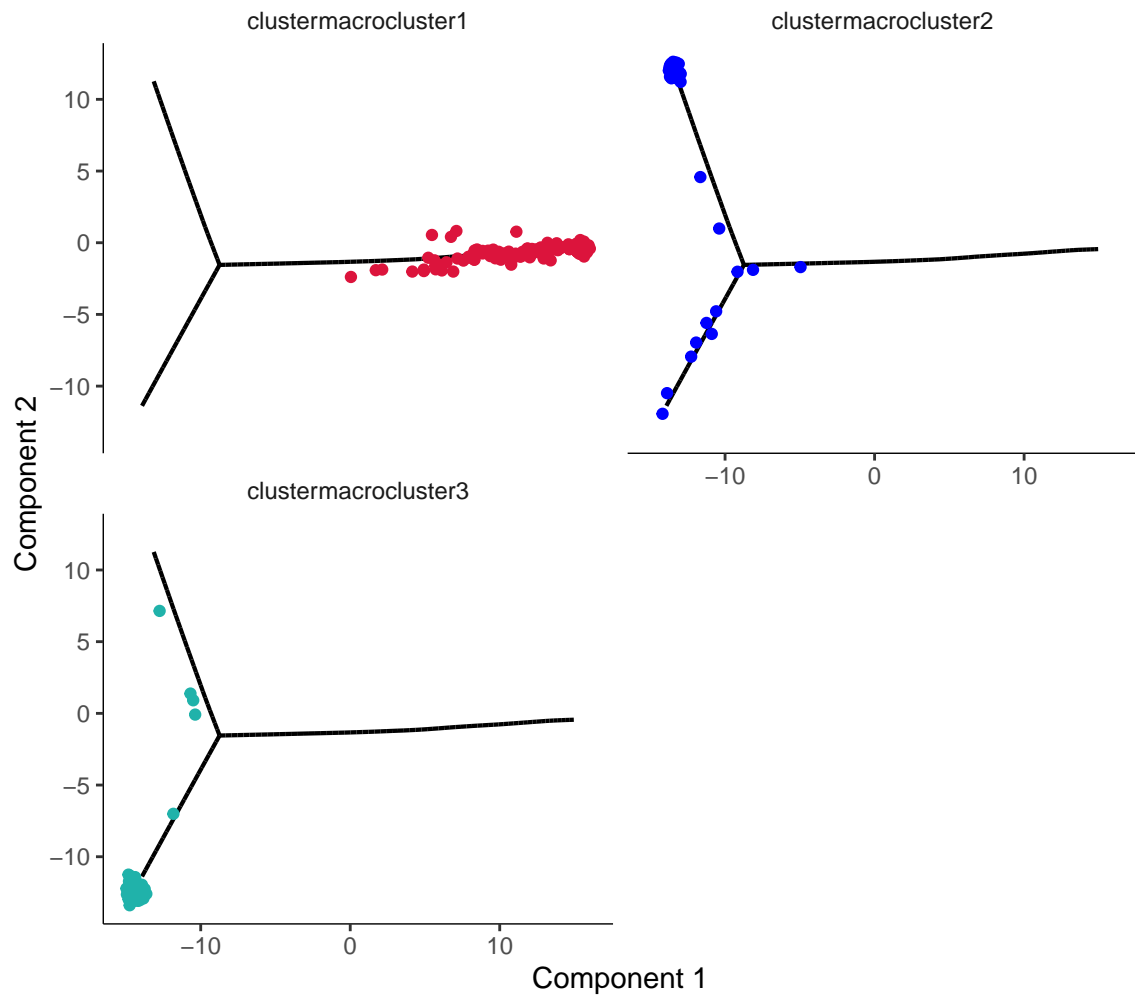

Supplement: Supplementary file 9 — Supplementary Figure 9. [file 41598_2024_52738_MOESM9_ESM.pdf]

● clustermacrocluster1 ● clustermacrocluster2 ● clustermacrocluster3

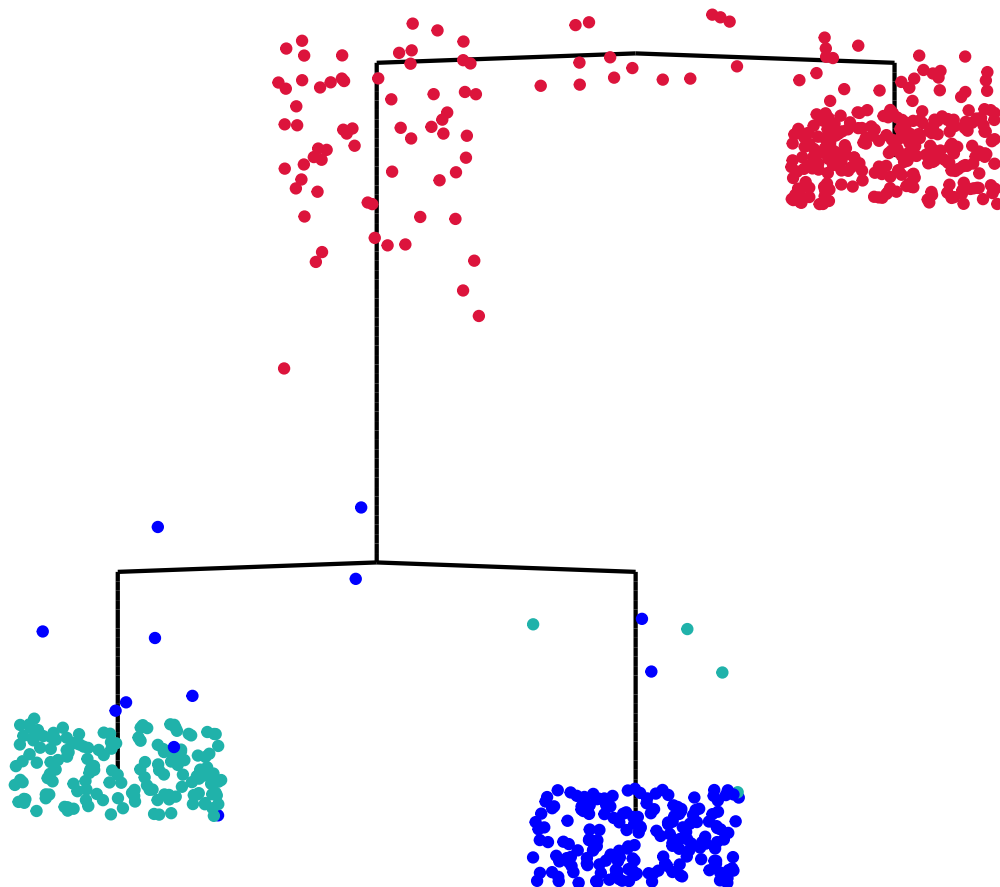

Supplement: Supplementary file 10 — Supplementary Figure 10. [file 41598_2024_52738_MOESM10_ESM.pdf]

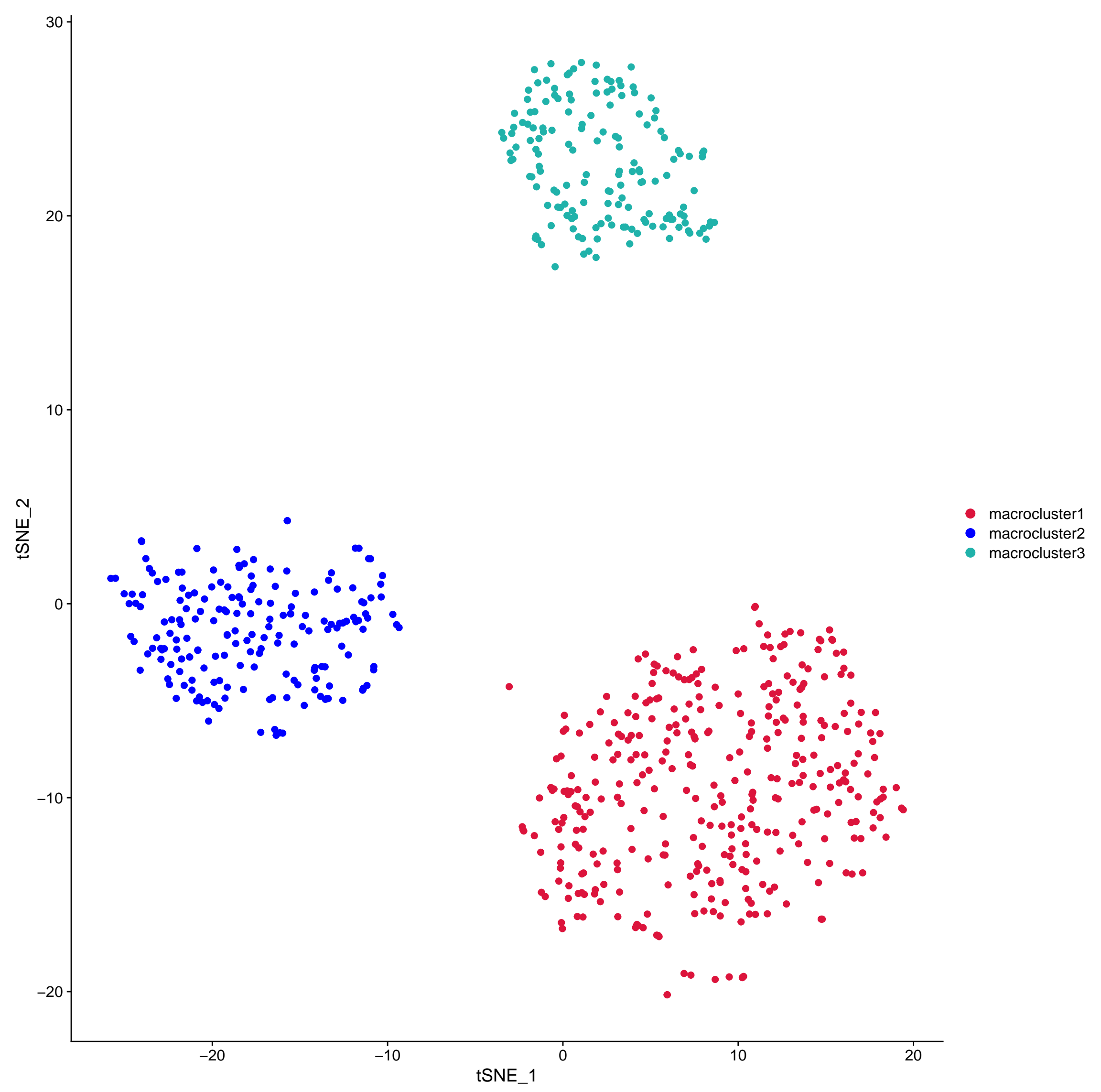

Supplement: Supplementary file 11 — Supplementary Figure 11. [file 41598_2024_52738_MOESM11_ESM.pdf]
